# High-Throughput Screening (HTS) and Hit Validation to Identify Small Molecule Inhibitors with Activity against NS3/4A proteases from Multiple Hepatitis C Virus Genotypes

**DOI:** 10.1371/journal.pone.0075144

**Published:** 2013-10-09

**Authors:** Hyun Lee, Tian Zhu, Kavankumar Patel, Yan-Yan Zhang, Lena Truong, Kirk E. Hevener, Joseph L. Gatuz, Gitanjali Subramanya, Hyun-Young Jeong, Susan L. Uprichard, Michael E. Johnson

**Affiliations:** 1 Center for Pharmaceutical Biotechnology and Department of Medicinal Chemistry and Pharmacognosy, University of Illinois at Chicago, Chicago, Illinois, United States of America; 2 Department of Pharmacy Practice, University of Illinois at Chicago, Chicago, Illinois, United States of America; 3 Department of Medicine, University of Illinois at Chicago, Chicago, Illinois, United States of America; Inserm, U1052, UMR 5286, France

## Abstract

Development of drug-resistant mutations has been a major problem with all currently developed Hepatitis C Virus (HCV) NS3/4A inhibitors, including the two FDA approved drugs, significantly reducing the efficacy of these inhibitors. The high incidence of drug-resistance mutations and the limited utility of these inhibitors against only genotype 1 highlight the need for novel, broad-spectrum HCV therapies. Here we used high-throughput screening (HTS) to identify low molecular weight inhibitors against NS3/4A from multiple genotypes. A total of 40,967 compounds from four structurally diverse molecular libraries were screened by HTS using fluorescence-based enzymatic assays, followed by an orthogonal binding analysis using surface plasmon resonance (SPR) to eliminate false positives. A novel small molecule compound was identified with an IC_50_ value of 2.2 µM against the NS3/4A from genotype 1b. Mode of inhibition analysis subsequently confirmed this compound to be a competitive inhibitor with respect to the substrate, indicating direct binding to the protease active site, rather than to the allosteric binding pocket that was discovered to be the binding site of a few recently discovered small molecule inhibitors. This newly discovered inhibitor also showed promising inhibitory activity against the NS3/4As from three other HCV genotypes, as well as five common drug-resistant mutants of genotype 1b NS3/4A. The inhibitor was selective for NS3 from multiple HCV genotypes over two human serine proteases, and a whole cell lysate assay confirmed inhibitory activity in the cellular environment. This compound provides a lead for further development of potentially broader spectrum inhibitors.

## Introduction

**Figure 1:**
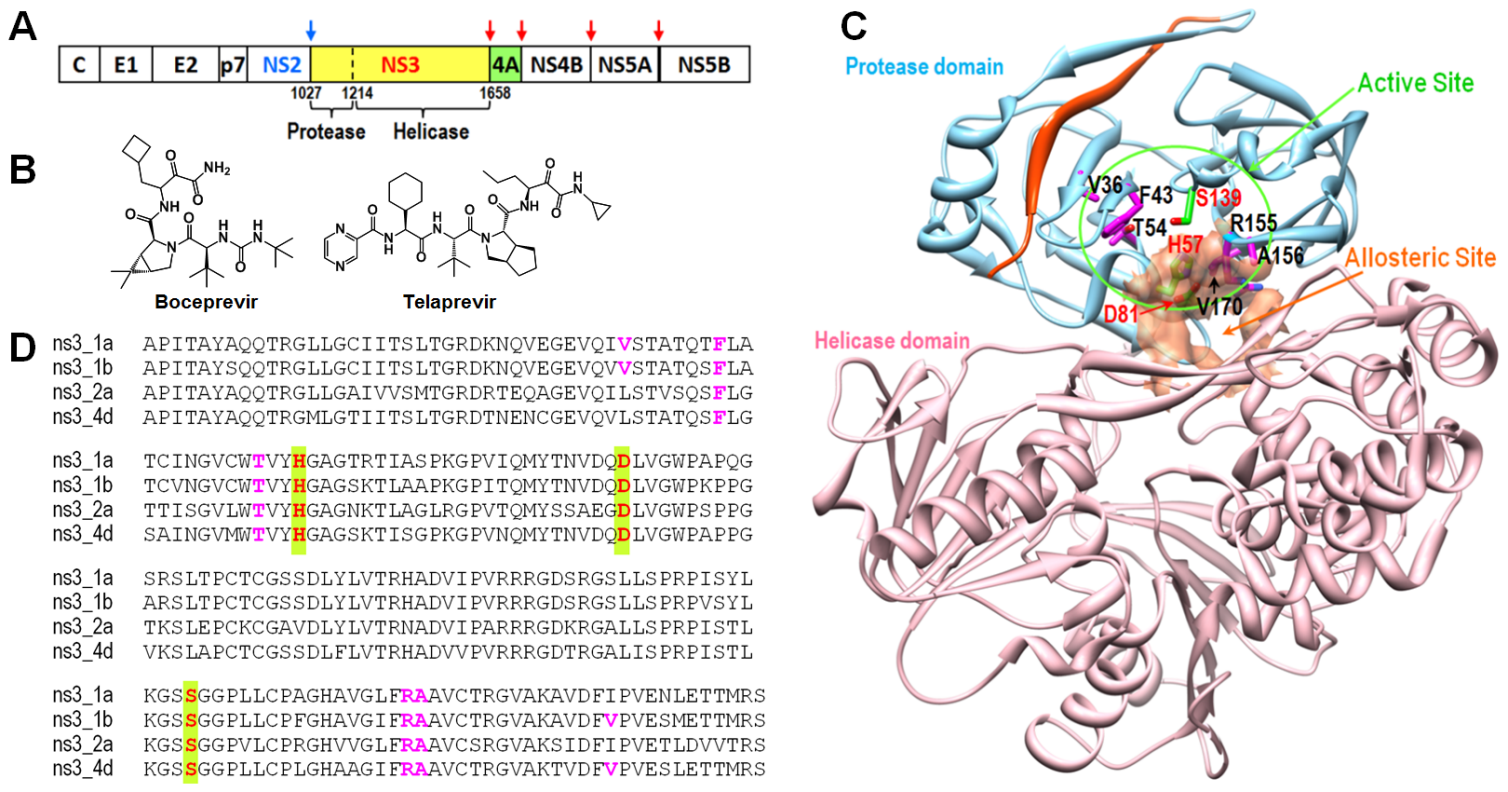
Background information and sequence alignment. (**A**) Schematic of the HCV polyprotein with cleavage sites of the two proteases, NS2 and NS3. (**B**) Structures of two FDA-approved NS3/4A inhibitors. (**C**) Structure of the NS3/4A serine protease, with the NS3 protease domain colored in cyan, and the co-factor NS4A (beta strand) shown in red. The active site residues, S139, H57 and D81, sit on the protein-protein interaction surface and are shown as stick figures in green. The amino acids prone to mutation in the binding site enabling drug resistance against both Telaprevir and Boceprevir are shown as stick figures in magenta (V36, F43, T54, R155 and A156). Images were prepared using Chimera v1.6.1, UCSF, 2012 [37]. (**D**) Sequence alignment of NS3 proteases from four HCV genotypes.

The Hepatitis C Virus (HCV) is a major cause of chronic liver diseases, hepatocellular carcinoma, and cirrhosis. It affects more than 180 million people, or about 3% of the world population [1], [2]. HCV is an enveloped virus with a positive single-stranded RNA-genome that is classified within the genus Hepacivirus of the family Flaviviridae [3]. The 9.6 kb HCV genome is translated into a polyprotein precursor and subsequently cleaved into four structural proteins (C, E1, E2, and p7) by the host cell, and into six non-structural proteins (NS2-NS5B) by two viral proteases, the NS2 cysteine protease and the NS3/4A serine protease (**Figure 1A**). NS2 cleaves at a single position between NS2 and NS3, and NS3/4A cleaves four subsequent downstream regions, releasing five proteins, NS3, NS4A, NS4B, NS5A, and NS5B [4]. NS3 is a multifunctional protein that contains a protease domain at the N-terminus and an RNA helicase domain at the C-terminus. It belongs to the trypsin/chymotrypsin protease super family, and the catalytic triad is made up of residues Ser139, His57 and Asp81 (**Figure 1C**) [4], [5]. In order for NS3 to function properly, NS4A is required as a cofactor and plays a role in proper positioning of the catalytic triad of NS3 and its substrate [5], [6]. Mutations to the catalytic residues of the NS3 protease prevented viral replication, thereby showing its essentiality. Therefore, NS3/4A is an attractive target for antiviral drug development against HCV [7].

Several large macrocyclic or linear peptidomimetic inhibitors have been reported, with the majority of these inhibitors developed by product peptide-based drug design followed by Structure-Activity-Relationship (SAR) studies to improve potency [8], [9]. Several NS3/4A inhibitors are in various phases of clinical trials, and there are two FDA approved NS3/4A inhibitors, VX 950 (generic name Telaprevir, brand name Incivek) [10] and SCH 503034 (generic name Boceprevir, brand name Victrelis) (**Figure 1B**) [11]. Most of these large inhibitors are competitive inhibitors that bind to the active site of the NS3 protease. Recently, Saalau-Bethell and coworkers reported the discovery of allosteric, small molecule inhibitors that bound to the interface of the NS3 protease and helicase (**Figure 1C**) [12]. These inhibitors did not have activity against the protease domain alone but were highly effective against the full-length NS3/4A, in which both the protease and helicase domains were present.

Direct-acting Antiviral Agents (DAA) such as inhibitors of NS3/4A, NS4B, NS5A, and NS5B have been helpful in combination therapy [13]. Unfortunately, resistance mutations have developed rapidly in NS3 against almost all currently developed inhibitors, including the two FDA approved drugs, significantly reducing the efficacy of these inhibitors. There are six common drug resistance mutation sites (V36, F43, T54, R155, A156, and V170) in the NS3 from genotype 1 generated against both Telaprevir and Boceprevir (see **Figure 1C and 1D**) [14]. Four additional mutation sites have developed against Boceprevir (Q41, V55, V158, and M175). In addition to the drug resistant mutants, there are more than 10 different HCV genotypes [15] that can be further categorized into sub-genotypes, further increasing the difficulty of drug design against HCV. These sub-genotypes are due to high mutation rates resulting from the lack of a proof reading function by HCV RNA polymerase NS5B [2], [16]. The HCV genotype 1b is the most prevalent worldwide, while genotype 1a is the most common in the United States. Genotype 2a is common in Japan and China, and genotype 4 is highly prevalent in the Middle East and central Africa [17]. The sequence identities of NS3 from genotypes 1a, 2a, and 4d are 90%, 69%, and 82%, respectively, as compared to the NS3 from genotype 1b (**Figure 1D**). The response rate of patients infected with genotype 1 (1a and 1b) to the current PEG-INTRON plus ribavirin standard therapy is only 40–50% while that of patients infected with genotypes 2 and 3 is approximately 80% [18], [19]. DAAs are recommended for the treatment of genotype 1 chronic HCV infection by the American Association for the Study of Liver Diseases [20]. Currently, the two NS3/4A serine protease inhibitors, Boceprevir and Telaprevir, have been approved as DAA for use in treatment of genotype 1 infections only. Combination therapy with NS3/4A protease inhibitors represents a major advancement in HCV treatment compared to traditional standard therapy. However, current NS3/4A inhibitors typically display variable activities across HCV genotypes, which will likely limit their broad usage against multiple genotypes. Combination therapy with NS3/4A protease inhibitors is also seriously hampered by the rapid development of drug-resistant mutants [21]. Therefore, next generation NS3/4A protease inhibitors with improvements in “pan-genotypic” activity and activity against drug-resistant mutants would be highly desirable as components of DAA cocktail therapy. In prior work, we have shown the feasibility of using computational approaches to discover NS3/4A inhibitors [22]. In this study, we used high-throughput screening (HTS) methods to discover novel small molecule inhibitors that have the potential both to inhibit NS3 enzymes from multiple HCV genotypes, and to inhibit several currently known drug resistant mutant NS3s from the genotype 1b with low micromolar activity.

## Materials and Methods

### Ethics Statement

N/A.

Experimental details of kinetic parameter determination and assay optimization for HTS are described in File S1.

### Preparation of Antimicrobial Focused Life Chemicals Library

A 25,000 compound drug-like chemical library was generated by selecting 18,750 (75%) compounds from the Life Chemicals antibacterial and antiviral activity targeted libraries. The remaining 6,250 compounds were selected from the general Life Chemicals screening collection, which were pre-filtered by the company to be ‘Rule of 5’ compliant (one exception tolerated). Additionally, a series of custom pre-filters (See **Table S1** in File S1) were applied to remove compounds with known reactive functionalities and/or toxicities prior to compound selection [23]. Molecular weight filters were also employed with a range of 150 to 650 Daltons. Finally, a diversity analysis was employed based upon 2D fingerprints to ensure that the most diverse selection of compounds was selected from each of the starting libraries.

### Tested Compounds

Compounds **1–3** were repurchased from Chembridge, compound **4** was repurchased from Sigma, compounds **5–8** were repurchased from Maybridge, and compounds **9–15** were repurchased from Life Chemicals. Compound purity was determined by NMR, HPLC, and/or LC/MS to be ≥95%. Each confirmed hit compound was repurchased at least twice in separate batches and tested again for activity. After being repurchased, compound **12**, our best hit, was repurified by HPLC, resulting in a purity of ≥99% (See File S1).

### Plasmid Construction and Purification of Wild-type and Mutant HCV NS3/4A Proteases

The full-length genes of wild-type HCV NS3 (HCV polyprotein residues 1027–1657) with a his-tag at the N-terminus and NS4A (residues 1658–1711) with no tag were cloned, co-expressed, and purified as described [24]. Eight NS3 drug resistant mutants (V36M, V36A, T54A, R155K, R155T, A156T, A156S, and 156V) were generated by mutagenesis using full-length, wild-type NS3-containing recombinant plasmid (pETDuet-1/FL NS3/4A) as a template. The over-expression and purification of all mutants were performed by the same method as the wild-type with minor modifications. Five mutants (V36M, R155K, A156T, A156S, and 156V) showed good enzyme activity while three mutants had very low enzyme activity compared to the wild-type.

### Primary HTS Screening

Three structurally diverse compound libraries, in-house (367), Prestwick (1,200), Maybridge (14,400), and the antimicrobial focused Life Chemicals library consisting of 25,000 compounds were screened against the full-length NS3/4A protease. The primary HTS assay was performed by a Tecan Freedom EVO 200 robot equipped with a Te-Mo 3×3 96-channel Liquid Handler dispenser and a 384-pin stainless steel pin tool (V&P Scientific) with a 200-nL capillary capacity. All assays were done in duplicate in black 384-well plates (Matrix Technologies). The NS3/4A enzyme (10 nM final concentration) was prepared in assay buffer (50 mM Tris, pH 7.6, 0.5% Chaps, 15% glycerol, 2 mM GSH, and 0.1 mg/mL BSA), and 30 µL was dispensed into wells. 200 nL of 10 mM compound (50 µM final concentrations) were then added and incubated for 5 minutes. Enzyme reactions were initiated with 10 µL of substrate Ac-DE-Dap(QXL520)-EE-Abu-ψ-[COO]AS-C(5-FAMsp)-NH_2_ (Anaspec) (1 µM final concentration), incubated for 6 minutes, and quenched by 10 µL of 10% SDS as a stop solution. Fluorescence intensity was monitored with a POLARstar OPTIMA microplate reader (BMG LABTECH). Each plate contained a total of 32 positive and 32 negative controls.

### Determination of Dissociation Equilibrium Constant (K_D_) by SPR

The full-length NS3/4A enzyme was prepared in a storage buffer (50 mM HEPES, pH 7.6, 500 mM NaCl, 1 mM DTT, 0.2% Triton X-100, and 20% glycerol) and immobilized on a CM5 sensor chip using standard amine-coupling at 25°C with running buffer HBS-P (10 mM HEPES, 150 mM NaCl, 0.05% surfactant P-20, pH 7.4) using a Biacore T100 instrument. Flow channels 1 and 3 were activated by 1-ethyl-3-(3-dimethylaminopropyl) carbodiimide hydrocholoride (EDC)/N-hydroxy succinimide (NHS) mixture, and the activated surface was blocked by ethanolamine (pH 8.5) as controls. The NS3/4A enzyme was diluted in 10 mM sodium acetate (pH 5.0), and immobilized to flow channel 4 after sensor surface activation with EDC/NHS with a 7 min injection followed by ethanolamine blocking on unoccupied surface area. NS3/4A immobilization level was ∼8,500 response units (RU). An unrelated reference protein (92 kDa) was also immobilized to flow channel 2 as another control to be compared with NS3/4A (74.6 kDa). Fifteen initial hit compounds were prepared as 10 mM DMSO stock solutions. Compound solutions with a series of increasing concentrations (0–200 µM at 1.5-fold dilution) were applied to all four channels at a 10 µL/min flow rate at 25°C. Sensorgrams were analyzed using Biaevaluation software 2.0.3, and response unit difference (ΔRU) values at each concentration were measured during the equilibration phase. Data were either single referenced with a blank (enthanolamine) or double referenced with both blank and reference protein RU values. SigmaPlot 11.0 was used to fit the data to a single rectangular hyperbolic curve to determine K_D_ values. The hyperbola function, y = y_max_·x/(K_D_+x), was used to plot response units and corresponding concentration, where y is the response, y_max_ is the maximum response, and x is the compound concentration.

### IC_50_ value Determination and Enzyme Omission Assay

All hit compounds from the HTS were cherry-picked and reanalyzed by continuous kinetic assay by hand for confirmation. For those that showed over 50% inhibition by a confirmation assay, IC_50_ values were measured by hand using the same assay conditions as the primary screen in triplicate. A series of compound concentrations (0 to 200 µM final concentration at 2-fold serial dilution) in 100% DMSO were prepared in a 384-well plate. 20 µL of enzyme solution was distributed to wells, and 0.5 µL of varying concentration of compounds were added and incubated for 5 minutes. The enzyme reaction was initiated by adding 5 µL of the substrate, and its activity was continuously monitored for 6 minutes. The IC_50_ values were calculated by fitting with the Hill equation (1), with OriginPro 8.5 (OriginLab, Inc.) where y is percent inhibition, x is inhibitor concentration, n is the slope of the concentration–response curve (Hill slope), and V_max_ is maximal inhibition from two to four independent assays.
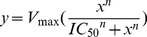
(1)


The enzyme omission assay was done by exactly the same method as IC_50_ determination, but without the NS3/4A enzyme in order to test for fluorescence signal interference by tested compounds.

### Reversibility of Inhibition

The reversibility of hit compounds was determined by dilution. The full-length NS3/4A enzyme complex was prepared as 20-fold (200 nM) of the assay concentration and was incubated with screened compounds at 20-fold IC_50_ value or slightly higher concentration for 30 minutes at room temperature in the same assay condition as the primary screen in a final volume of 100 µL. The concentration of enzyme and testing compound was optimized to yield at least 95% inhibition before dilution. The NS3/4A enzyme with the same volume of DMSO in place of each compound was also prepared as a control. NS3/4A activity was measured in the same way as the IC_50_ measurements. Then enzyme-inhibitor solution was diluted 25-fold and 50-fold and incubated for 30 minutes before measuring the percent recovery of the enzyme activity. All reversibility assays were done in triplicate.

### Inhibition Assay with Other HCV Genotypes and NS3 Drug-resistant Mutants

The inhibitory activity of the final hit compounds from HTS were tested against three other HCV genotypes (1a, 2a, and 4d), along with a control (genotype 1b) and five NS3 drug-resistant mutants. Eight full-length NS3 mutants (V36M, R155K, A156T, A156S, 156V, V36A, T54A, and R155T) were generated by mutagenesis and purified, and the three HCV NS3/4A protease genotypes were purchased from Anaspec. Unfortunately the enzyme activities of the three mutants V36A, T54A, and R155T were much less than that of the rest of the mutants and were insufficient for testing, therefore mutant studies were done with the five active mutants. Continuous kinetic assays were performed against all hit compounds in the same way as the wild-type NS3/4A. As a control, full-length NS3/4A (genotype 1b) was tested under the same conditions for direct comparison for each plate.

### Type of Inhibition

Full-length NS3/4A activity was monitored in the same way as the primary screening with varying concentration of both inhibitor compounds and substrate (0–8 µM). The concentration of compounds varied from 0 to 20 µM at 1.5-fold dilution factor. The data were fit to equations 2–5 using SigmaPlot Enzyme Kinetics Module 1.3 in order to determine the best fit mechanism for each compound. Mechanism of inhibition and kinetic parameters were determined from the best fit equations among these four equations, equation 2 for competitive inhibition, equation 3 for non-competitive inhibition, equation 4 for uncompetitive inhibition, and equation 5 for mixed-type inhibition.
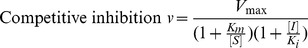
(2)

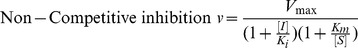
(3)

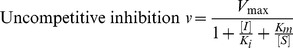
(4)

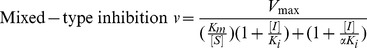
(5)where *v* is the reaction rate, V_max_ is the maximum rate of the reaction, K_m_ is the Michaelis-Menten constant for substrate, [S] is the substrate concentration, [I] is the inhibitor concentration, K_i_ is the dissociation constant of the inhibitor I to the free enzyme and αK_i_ is the dissociation constant for the inhibitor I to the ES complex.

### Microsomal Stability Assay

A typical incubation mixture (100 µL total volume) for the metabolic stability studies contained 1 µM (final concentration) test compounds, 0.5 mg/mL microsomal protein (pooled Balb/c mouse liver microsomes prepared as described previously [25]), 100 mM Tris-HCl buffer (pH7.4), and NADPH-generating system (5 mM isocitric acid, 0.2 unit/mL isocitric acid dehydrogenase, 5 mM magnesium chloride, 1 mM NADP^+^). After pre-incubation at 37°C for 5 minutes, the reactions were started by addition of NADP^+^ and further incubated for another 0, 5, 10, and 20 minutes. For control experiments, NADPH and/or liver microsomes were omitted from these incubations. The reactions were terminated by adding 100 µL ice-cold acetonitrile containing phenytoin (1 µM) as an internal standard and kept on ice for 30 minutes, followed by centrifugation at 16,100 *g* for 15 minutes to obtain the supernatant. Aliquots (5 µL) were then analyzed for substrate disappearance using liquid chromatography-tandem mass spectrometry (Agilent 1200 HPLC interfaced with Applied Biosystems Qtrap 3200) equipped with an electrospray ion source. Chromatographic separation was carried out with a Waters XTerra MS C18 column (2.1×50 mm, 3.5 µm; Agilent Technologies, Santa Clara, CA). The mobile phases consisted of solvent A (0.1% (v/v) aqueous formic acid) and solvent B (acetonitrile). A 250 µL/min flow rate gradient was developed for each test compound over 10 minutes (**Table S2** in File S1). Mass detection of test substrates and internal standard were followed in a positive ion mode by examining multiple reaction monitoring (MRM) pairs. Compound-dependent mass parameters were optimized by infusion method and summarized in **Table S2** in File S1. The spraying needle voltage was set at 5000 V. Curtain gas was set at 20; gas 1 and gas 2 were set at 45 and 50, respectively; collision assisted dissociation gas was at medium; and the source heater probe temperature was at 500°C. The test compounds were quantified by comparing the ratio of ion currents obtained for the substrates and an internal standards calibration curve. Data acquisition and processing were accomplished using Analyst software (version 1.4.1; Applied Biosystems). Apparent half-lives (t_1/2_) for the disappearance of parent drugs were calculated from the pseudo-first-order rate constants (k_e_) obtained by linear regression of plots of log [drug remaining] versus time (GraphPad Prism 5 software, La Jolla, CA) using the equation: *t_1/2_ = *0.693*/k_e_*.

### HCV Subgenomic Replicon Cells and Whole Cell Lysate Inhibition Assay

Huh7-1 cells (also known as Huh7/scr cells) were obtained from F.V. Chisari (The Scripps Research Institute, La Jolla, CA) [26]. Clone B HCV sub-genotype 1b Huh7 cells were obtained from the NIH AIDS Research and Reference Reagent Program and have been previously described [27]. Huh7 cells stably replicating the sub-genotype 2a HCV JFH-1 replicon were generated as previously described [28]. All cells were cultured in complete Dulbecco’s modified Eagle’s medium (DMEM) supplemented with 10% fetal bovine serum, 100 units/mL penicillin, 100 mg/mL streptomycin, and 2 mM l-glutamine. HCV replicon cells were maintained in 500 µg/mL Geneticin (Invitrogen). For NS3 inhibition analysis, lysates from parental Huh7 cells and HCV replicon cells were harvested on ice in pre-chilled FRET cell lysis buffer (50 mM Tris-HCl, pH 7.5; 150 mM NaCl, 2 mM EDTA, 1.25% Triton X-100). Lysates were brought to room temperature and mixed 1∶1 with 2× FRET assay buffer (100 mM Tris-HCl, pH 7.5, 300 mM NaCl, 4 mM EDTA, 2.5% Triton X-100, 10 mM GSH, and 4 µM substrate). Fluorescence intensity was continuously monitored for 1 hour, and IC_50_ values were determined in the same way as biochemical assays.

### Inhibitor Selectivity Assay

To test for selectivity, two human serine proteases, Trypsin and Chymotrypsin (Sigma), were tested with the top two hit compounds from HTS using a fluorometric assay. The fluorogenic substrates used in this study were N-benzoyl-L-Arg-7-amido-4-methylcoumarin (Sigma) and N-succinyl-Ala-Ala-Phe-7-amido-4- methylcoumarin (Sigma) for trypsin and chymotrypsin, respectively. All assays were performed in 384-well black plates (Corning) in a total volume of 24 µL of PBS (pH 7.4) buffer containing 0.01% Triton, 0.1 mg/mL BSA, and 2 mM GSH in triplicate. A series of compound concentrations (0 to 200 µM final concentration at 2-fold serial dilution) in 100% DMSO was prepared in a 384-well plate. 20 µL of trypsin (0.01 mg/mL) and chymotrypsin (0.0001 mg/mL) solutions were distributed into wells, and 0.5 µL of varying concentration of compounds were added and incubated for 10 minutes. The enzyme reaction was initiated by adding 5 µL of the substrate (50 µM final concentration), and fluorescence intensity was continuously monitored at excitation/emission wavelengths of 350 nm/460 nm for 10 minutes.

### Molecular Docking

Molecular dockings were performed using the GOLD v5.0.1 program [29]. The crystal structures of the full-length HCV NS3/4A protease-helicase (genotype 1b) in complex with a macrocyclic protease inhibitor (PDB code 4A92) and HCV NS3/4A protease domain (genotype 1a) complexed with a macrocyclic ketoamide inhibitor (PDB code 2GVF) were prepared using the Protein Preparation Wizard. The active site was defined with a 10 Å radius around the ligand present in the crystal structure. The compounds were prepared using LigPrep [30]. The OPLS2005 force field was used for geometric optimization, and all possible ionization and tautomeric forms were created at pH 7±1 using EPIK [31]. The best scoring pose for each compound from each of the 100 independent genetic algorithm runs was saved for further analysis.

## Results and Discussion

### High-throughput Screening and Hit Validation

**Figure 2:**
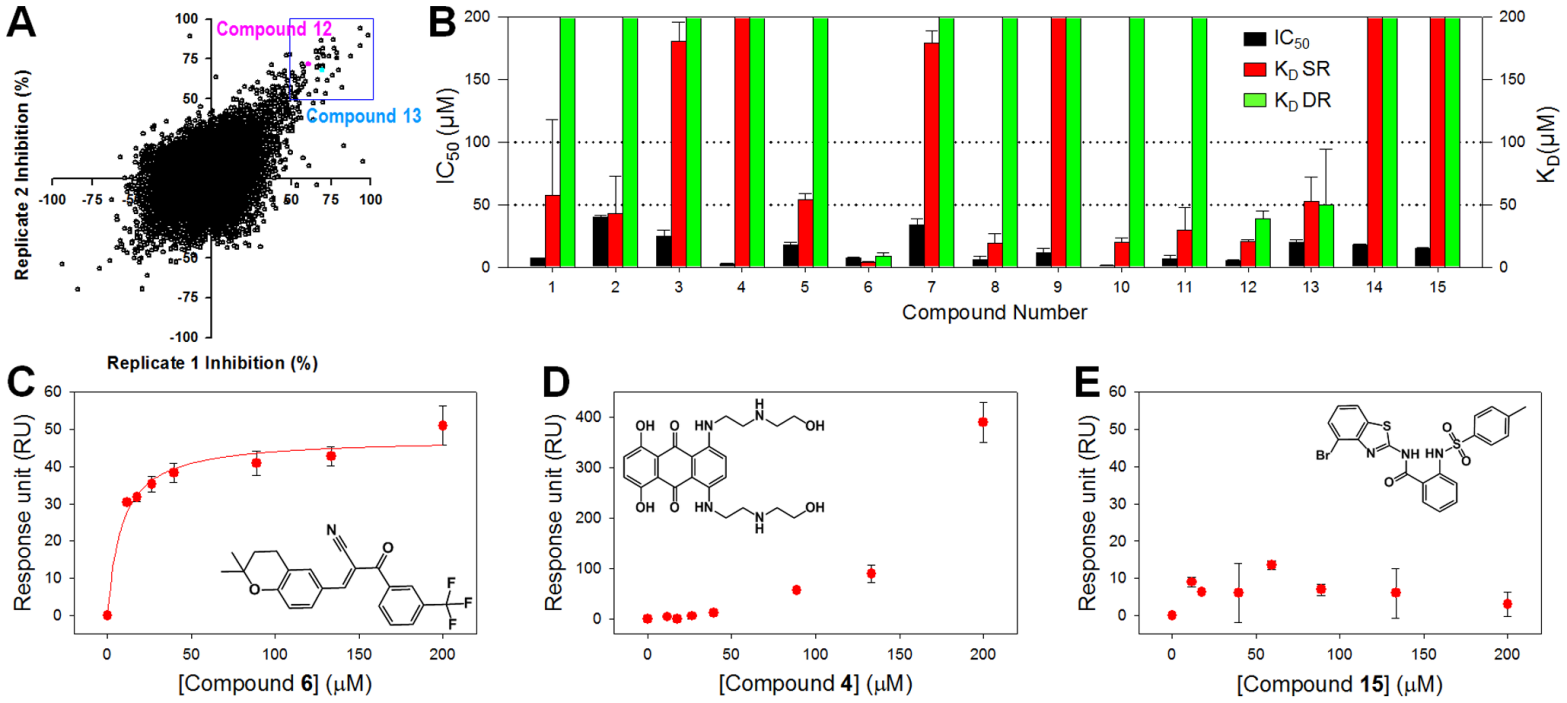
Primary HTS results and SPR validation binding assay results. (**A**) Replicate plot of the screening of 40,967 compounds from structurally diverse in-house, Prestwick FDA-approved drug, Maybridge, and Life Chemicals libraries. The blue box indicates hit compounds with over 50% inhibition at 50 µM compound concentration. Initial percent inhibitions of the two confirmed hits are shown in pink (Compound **12**) and cyan (Compound **13**). (**B**) Bar graphs of IC_50_ values and the dissociation equilibrium constants (K_D_) of 15 initial hit compounds. K_D_ SR is single referenced K_D_ values with a blank channel immobilized with a small molecule ethanolamine, whereas K_D_ DR represents double referenced with ethanolamine and an unrelated reference protein (*B. anthracis* PyrC, ∼95 kDa as a dimer) immobilized in two different channels. All data were normalized for immobilization levels of target and reference proteins. Bars that reached the top of the graph represent K_D_ values of over 200 µM (no binding). (**C**) The fitting curve of compound **6** with a single rectangular hyperbolic equation (See methods). The determined K_D_ of compound **6** was 8.3±1.9 µM, similar to its IC50 value (7.6±0.6 µM). (**D**) Response units of compound **4** (mitoxantrone from the Prestwick) at a series of increasing concentrations (0–200 µM), showing a non-specific binding pattern. (**E**) Response units of compound **15** at various concentrations (0–200 µM), showing lack of binding to NS3/4A.

Four structurally diverse compound libraries (an in-house collection of 367 compounds, the 1,200 Prestwick FDA-approved drugs library, a 14,400 Maybridge diversity set, and an antimicrobial/antiviral focused Life Chemicals library of ∼25,000 compounds) were screened against the full-length NS3/4A by HTS. Typically, HCV NS3 compound screening uses either the NS3 protease domain with an NS4A core peptide or a single-chain NS4A-NS3, which is a modified version of NS3 in which only part of the NS4A core peptide (fourteen residues, 21–34) is connected to the N-terminus of NS3. The studies presented here differed from this by our use of co-expressed and purified full-length NS3 and NS4A proteins. All screened compound libraries and statistical parameters of hit compounds are summarized in **Table 1**. The primary screens of the in-house, Prestwick, Maybridge, and Life Chemicals libraries were performed in duplicate, with Z’-factors of 0.75±0.08, 0.67±0.10, 0.62±0.05, and 0.65±0.18, respectively. The Z’-factor represents the signal-to-background ratio combined with the coefficient of variation of the background. In order for an HTS to be considered as a good quality assay, the Z’-factor should be between 0.5 and 1.0, with higher values better. The replicate plot is shown in **Figure 2A**.

**Table 1:** Statistical parameters of all screened compounds from four libraries.

Library	Number ofcompounds	Primary hits (≥50%inh/IC_50_≤50 µM)	Reordered	Hits (IC_50_≤50 µM/Reversible hits)	Enzymeomission assay	Bindingconfirmed by SPR	Final hitrate (%)
In-house	367	13/9	9	5/3	3	0	0
Prestwick	1,200	8/2	2	2/1	1	0	0
Maybridge	14,400	22/11	11	7/4	4	1	0.007
Life Chemicals	25,000	59/28	19	11/7	5	2	0.008

Final hit rates are calculated only with hits confirmed by SPR.

Primary screening by end-point fluorescence-based enzymatic assays resulted in a total of 102 hit compounds with over 50% inhibition at 50 µM compound concentration. The inhibitory activities of 50 compounds were confirmed by continuous fluorescence assays and IC_50_ determination with cherry-picked compounds. After visual inspection, 41 compounds were reordered, only 25 of which still showed IC_50_ values below 50 µM. Of these 25 compounds, 15 were reversible. Enzyme omission assays showed two of these compounds to be false positives due to compound interference with fluorescence. In order to further eliminate false positives and validate true hit compounds, an orthogonal binding assay by surface plasmon resonance (SPR) was performed for 15 potential hit compounds. The two fluorescence signal interfering compounds were included in SPR analysis to further analyze their behavior among other hits. The dissociation equilibration constants (K_D_) of eleven compounds were successfully determined by SPR, indicating direct binding to NS3/4A with a single reference using blank immobilization with a small molecule, ethanolamine (**Figure 2B and 2C**). However, four compounds either did not bind or bound non-specifically to the NS3/4A enzyme. Specifically, three compounds (**4**, **9**, and **14**) showed non-specific binding patterns with NS3/4A (example curve for **4** shown in **Figure 2D**), and **15** did not bind to the enzyme (**Figure 2E**). Surprisingly, only three compounds (compound **6**, **12**, and **13**) exhibited specific binding to the NS3/4A when all data was double referenced with an unrelated reference protein, indicating that some of these compounds unfortunately bound to the reference protein as well as the NS3/4A enzyme. The majority of nonspecific binders were either strong Michael accepters or rhodanine compounds. Although **6** was reversible and showed specific interaction to the NS3/4A, the structure of this compound is not attractive as it is a Michael Acceptor. Therefore, only two compounds (**12** and **13**) were considered appropriate for progressing to the next steps.

### Type of Enzyme Inhibition

**Figure 3:**
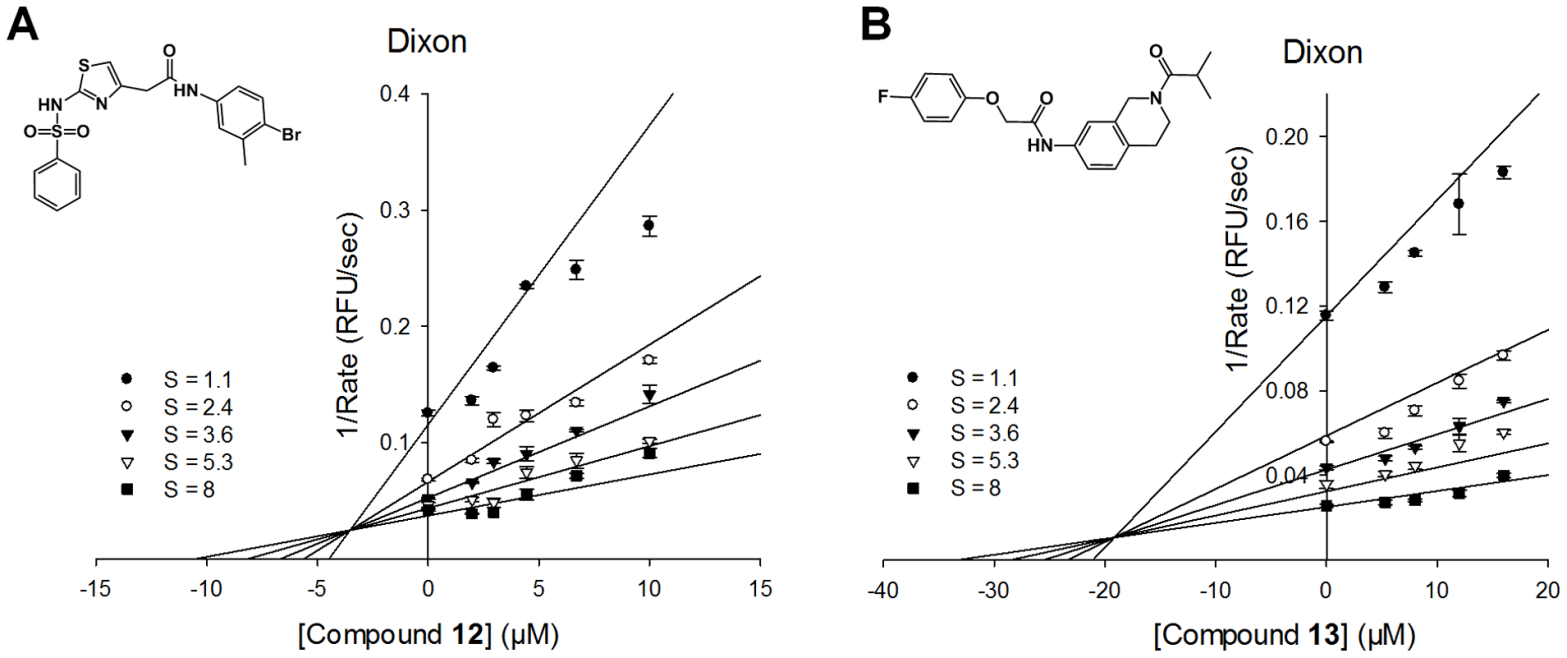
Mechanism of enzyme inhibition of 12 and 13. Dixon plot for competitive inhibition of Compound **12** (**A**) and Compound **13** (**B**) with respect to the substrate Ac-DE-Dap(QXL520)-EE-Abu-ψ-[COO]AS-C(5-FAMsp)-NH_2_. Determined *K_i_* values of Compound **12** and **13** were 3.5 µM and 19.1 µM, respectively. A known competitive inhibitor BILN-2061 was used as a control. Four equations (See Experimental section) in SigmaPlot Enzyme Kinetics Module 1.3 were used to fit the experimental data. The competitive inhibition model was the best fit for both compounds and for the control.

With the recent discovery of allosteric inhibitors of NS3/4A that bind to the interface between the protease and helicase domain [12], it was important to determine whether our newly discovered small molecule inhibitors bind to the protease active site or to the interface allosteric site. The former would be competitive inhibitors with respect to the NS3 protease substrate, whereas the latter would show other types of inhibition that would indicate allosteric site binding and inhibition. We thus investigated the mechanism of inhibition of the two newly identified compounds **12** and **13**. Kinetic studies for each compound were performed with the enzyme-inhibitor complexes and varying substrate concentrations. Data were fit to each of four equations (see Method) and three plots (Michaelis-Menten, Lineweaver-Burke, and Dixon), all of which were analyzed using SigmaPlot Enzyme kinetics Module 1.3. Akaike Information Criterion corrections (AICc) for sample size values were used to determine the best fit equation following SigmaPlot instructions, along with standard errors of the parameter estimates [32]. The best fit equation had the lowest AICc value, with a minimum of 2 AICc units difference from the next lowest. Both **12** and **13** were determined to be competitive inhibitors with respect to the substrate for the active site, with K_i_ values for **12** and **13** of 3.5 µM and 19.1 µM, respectively (**Figure 3**). A known competitive inhibitor BILN-2061 was also analyzed similarly, as a control.

We further compared inhibitory activities of **12** with the full-length NS3/4A and the NS3/4A protease domain alone to confirm whether this compound bound to the protease active site or to the interface. The former should have inhibitory activity against the protease domain alone as well as full-length NS3/4A, whereas the later should only show activity against full-length NS3/4A. Inhibitory activity of compound **12** was determined along with BILN-2061, which binds to the active site of NS3 protease as a control (**Table 2**). Compound **12** showed comparable IC_50_ values against both NS3 protease domain alone (9.3 µM) and full-length NS3/4A (2.2 µM), similarly to BILN-2061, providing evidence that these inhibitors bind to the active site of the NS3 protease, rather than the allosteric site. Hence, our newly discovered compound is one of the first reported non-peptidic small molecule competitive inhibitors that bind to the active site of the NS3 protease.

**Table 2:** Comparison of the inhibitory activities of 12 with a control.

Compound	IC_50_ (µM)		LE
	Full-length NS3/4A	NS3 protease domain	(kcal/mol/heavy atom)
BILN-2061	0.0046±0.0005	0.0017±0.0001	
**12**	2.2±0.4	9.3±1.6	0.29

IC_50_ values were determined by measuring fluorescence intensity at a series of compound concentrations with the full-length NS3/4A and NS3/4A protease domain only for comparison from three independent assays. Ligand efficiency (LE) was calculated with IC_50_ values from the full-length NS3/4A.

### Activity Testing Against NS3/4A Enzymes from Four HCV Genotypes

**Figure 4:**
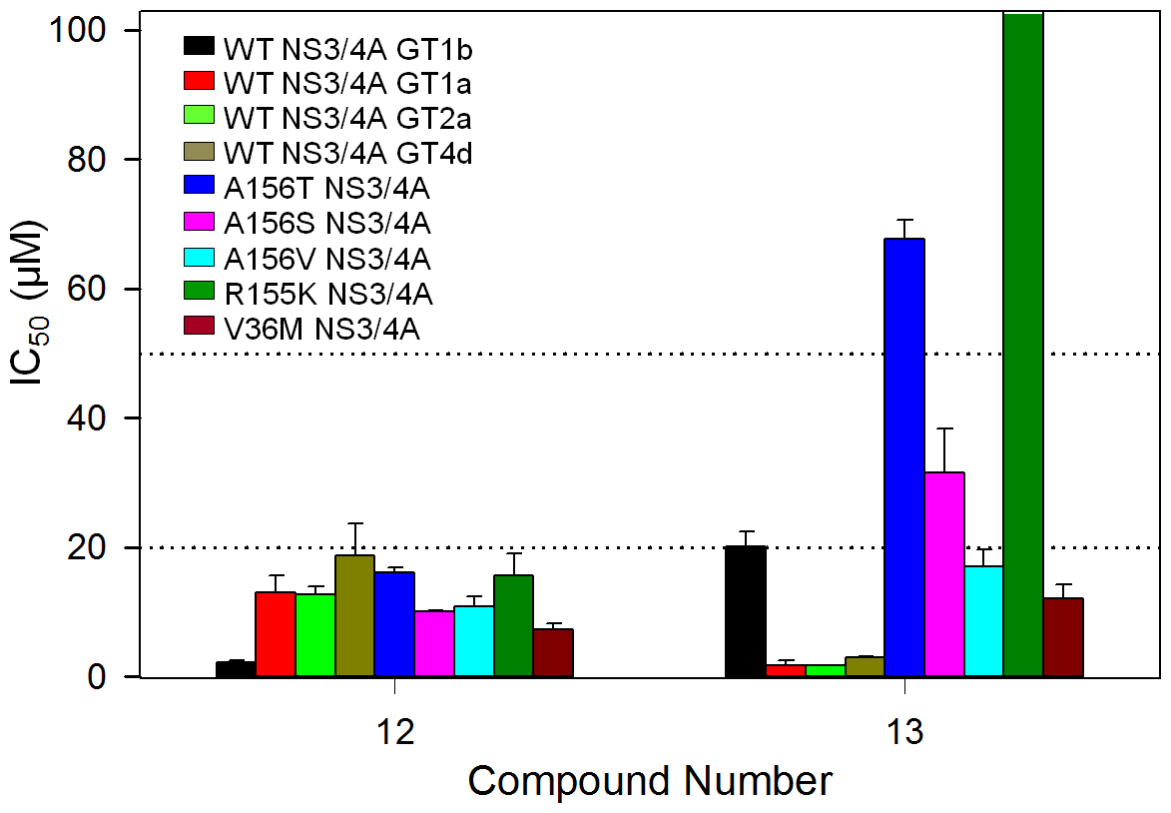
IC_50_ value comparisons of two confirmed hit compounds. Bar graphs are shown with IC_50_ values of two identified compounds against NS3/4As from four HCV genotypes, genotypes 1a (GT1a), 1b (GT1b), 2a (GT2a), and 4d (GT4d), and five mutants from genotype 1b. IC_50_ determination was done in triplicate with a total of 32 positive and 32 negative controls in a plate. IC_50_ values were calculated by fitting the data to the three parameter Hill equation with OriginPro 8.5 (See Methods). Bars that reached the top represent IC_50_ values of over 100 µM (no inhibitory effect).

The high mutation rate of HCV has resulted in the generation of several different HCV genotypes that exhibit varying responses to the current standard treatment. Our initial screen was performed against the full-length NS3/4A from genotype 1b; however, we also tested two confirmed hits (**12** and **13**) against the NS3/4A protease domains from three additional HCV genotypes (1a, 2a, and 4d). The two compounds showed varying degrees of inhibition against enzymes from the four genotypes (**Figure 4**). Compound **12** showed IC_50_ values below 20.0 µM against NS3/4A enzymes from all four genotypes with the best activity against genotype 1b. On the other hand, compound **13** was approximately 12-fold more effective (1.7 µM) against NS3/4A from genotype 1a than that (20.2 µM) of genotype 1b NS3/4A. Overall, two new structurally differing and promising compounds (**12** and **13**) showed inhibitory activity with IC_50_ values below 20 µM against NS3/4A from all four HCV genotypes.

**Figure 5:**
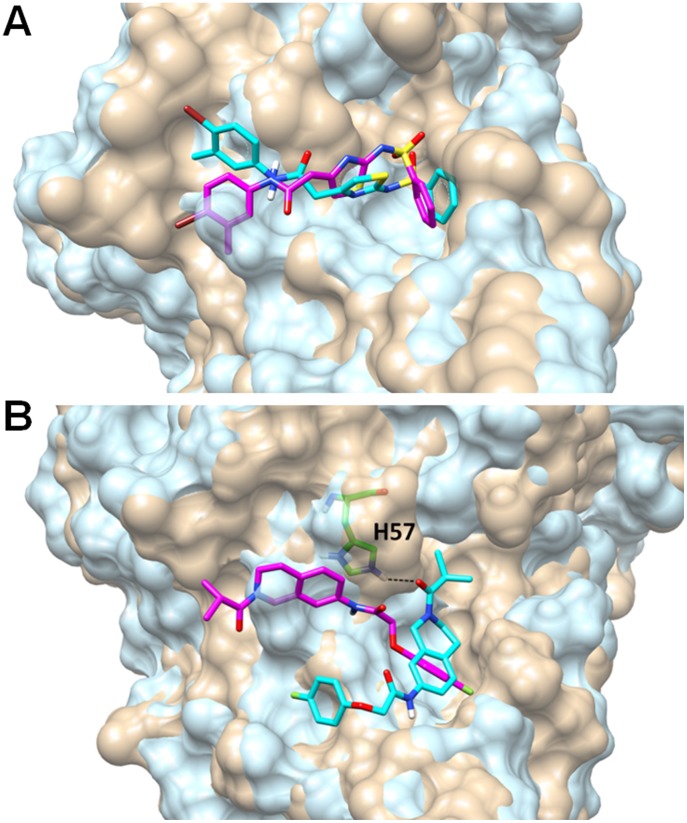
Comparison of the docking poses with NS3 proteases from sub-genotype 1a and 1b. (**A**) Docking poses of **12** colored in pink with the sub-genotype 1b NS3 protease (colored in tan). Compound **12** is colored in cyan when it was docked with the sub-genotype 1a NS3 protease (colored in cyan). (**B**) Docking poses of compound **13** colored in cyan with sub-genotype 1a (also colored in cyan). The histidine 57 residue is shown in green, and a hydrogen bond with **13** is shown as a black dotted line. Compound **13** is colored in pink when it was docked with sub-genotype 1b (colored in tan) NS3 proteases. Figure prepared with Chimera v1.6.1, UCSF, 2012 [37].

We hypothesized that the variation in inhibitory activity is due to differences between the protease active sites among the enzymes from different genotypes and the different binding modes of the inhibitors. The binding pockets of NS3 from sub-genotype 1a and 1b are quite different, despite the overall high sequence conservation between the two (pairwise sequence identity, 90%). As we noted recently [22], the binding site of NS3 1a is more open, narrower, less complex, and less favorable for binding small molecules when compared to the binding site of NS3 1b. Our docking studies suggest that inhibitors with cross-genotypic activity maintain similar binding modes against NS3/4A from different genotypes. For example, **12** maintains a similar docked binding mode in the active site of enzymes from both sub-genotypes 1a and 1b, with minor differences to adapt to the differing shapes of the active sites (**Figure 5A**). On the other hand, **13** showed greater activity against NS3/4A enzymes from sub-genotype 1a compared to 1b. Docking results suggest that these compounds bind somewhat differently in the pockets of the two enzymes (**Figure 5B**). The docking poses of **13**, which exhibited a 10-fold decrease in IC_50_ values from 1b to 1a, against the different sub-genotype enzymes differed significantly. From **Figure 5B**, it can be seen that **13** forms a hydrogen bond with the catalytic residue His57 in the enzyme from sub-genotype 1a, while it does not form any hydrogen bond interaction with the enzyme from sub-genotype 1b. This additional binding interaction may explain the enzymatic selectivity of this compound.

### Activity Testing Against NS3/4A Drug-resistant Mutants

**Figure 6:**
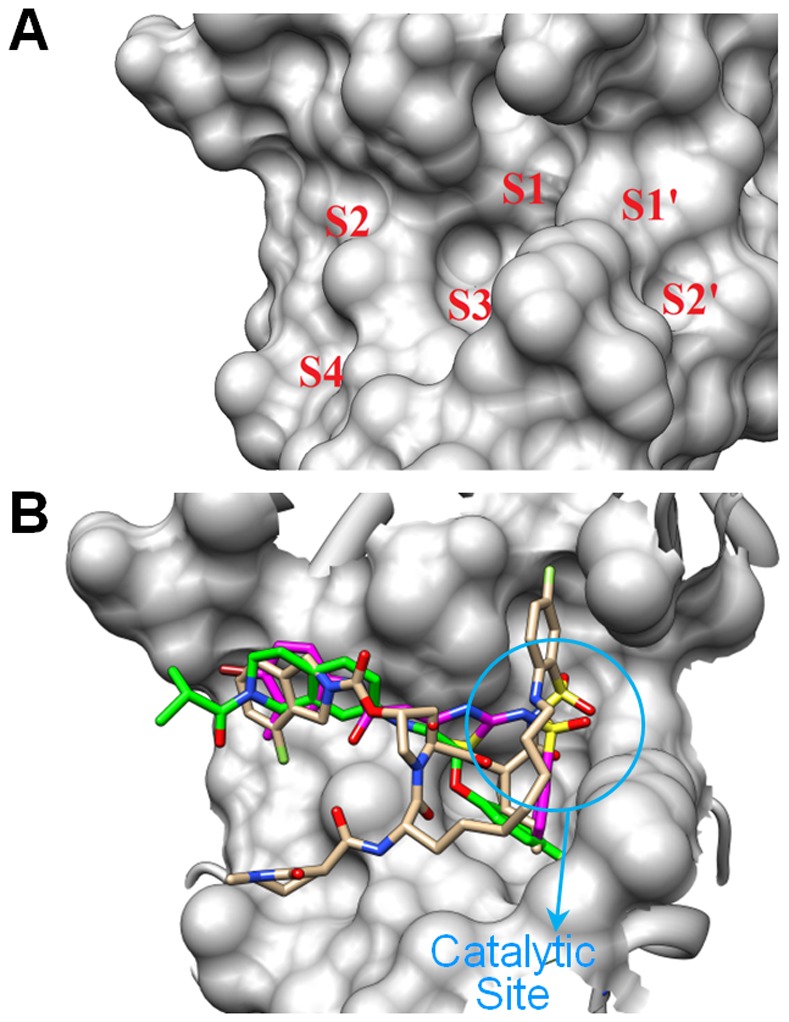
Substrate binding sites and docking studies. (**A**) The surface of the NS3 protease substrate binding sub-sites. The S4-S2’ binding pockets are labeled in red. (**B**) Active-site view of docked **12** (colored in green) and **13** (colored in magenta) overlaid with a known macrocyclic inhibitor ITMN191 (colored in tan) from crystal structure (PDB code: 4A92). Figure prepared with Chimera v1.6.1, UCSF, 2012 [37].

One of the most critical problems with the currently approved NS3/4A protease inhibitors and inhibitors in clinical trials is the development of drug-resistance conferring mutations. There are two residues, R155 and A156 that are susceptible to the development of drug-resistant mutations against almost all known NS3/4A inhibitors. A recent study showed that inhibitors that can fit within the substrate envelope are less likely to be affected by drug resistance mutations [14]. This is because functional mutants are still able to bind the natural substrate, but any inhibitor volume protruding from the substrate envelope, especially at the S1 and S2 sites (**Figure 6A**), will be susceptible to mutations that affect the binding affinity of the inhibitors [14]. Boceprevir and two other NS3/4A inhibitors (ITMN-191 and TMC-435) in clinical trials are susceptible to mutations at both positions R155 and A156. These compounds interact with the mutations at the S2 sites, where their protruding volume from the substrate envelope is large [14]. Two hit compounds were tested against five common drug-resistant mutants (A156T, A156S, A156V, R155K, and V36M) of full-length NS3/4A from genotype 1b (**Figure 4**). We discovered that compound **12** maintained IC_50_ values at or below 20 µM against both wild-type and all five drug-resistant mutants, while compound **13** decreased in activity against two mutants (A156T, A156S) and completely lost activity against R155K. Two NS3 mutants, A156V and V36M, were less affected, maintaining inhibitory activity below 20 µM, similar to that against the wild-type NS3/4A. We believe that this retention of inhibitory activity against the common resistance mutations is quite promising from the perspective of the development potential of these or similar compounds.

### Inhibitor Specificity and Microsomal Stability

HCV NS3/4A is a serine protease that belongs to the trypsin/chymotrypsin protease superfamily [33]. It has a catalytic motif similar to that of many human serine proteases, and hence it is important to develop selective inhibitors for HCV NS3/4A. Sequence alignment and 3D alignment of the active sites of 14 human serine proteases in comparison with HCV NS3/4A were analyzed (**Figure S1**–**2** in File S1). Almost all 14 human serine proteases showed similar active site structures, but the HCV NS3/4A active site was very different. We tested the selectivity of the two inhibitors, **12** and **13**, against two human serine proteases, trypsin and chymotrypsin. These two inhibitors showed IC_50_ values greater than 100 µM against both trypsin and chymotrypsin. Therefore, these two inhibitors were selective for HCV NS3/4A over the two human serine proteases (**Table 3**).

**Table 3:** Selectivity and microsomal stability of selected compounds.

Compound	Selectivity (IC_50_ (µM))			Microsomal stability
	HCV NS3/4A	Trypsin (% Inh at100 µM)	Chymotrypsin (% Inh at100 µM)	t_1/2_ (min)
**4**	3.1±0.1	>100 (22%)	>100 (0%)	25.4
**12**	2.2±0.4	>100 (28%)	>100 (19%)	21.7
**13**	20.2±2.2	>100 (5.1%)	110±32	ND

Percent inhibitions at 100 µM are shown in parenthesis for both Trypsin and Chymotrypsin. All IC_50_ values and percent inhibition are from three to four independent assays. ND, not determined.

Microsomal stability was analyzed for two compounds (compounds **4** and **12**). Compound concentration for both compounds decreased in microsomes supplemented with NADPH, a cofactor for cytochrome P-450 (CYP) and flavin-containing monooxygenase (FMO). The compound concentration did not decrease in microsomal reactions lacking the cofactor, indicating that these compounds are likely oxidized by CYP and/or FMO in our system. Our best lead compound, compound **12**, showed microsomal stability of 22 minutes, which is comparable to mitoxantrone (compound **4**, 25 minutes), a clinically used drug.

### SAR Analysis for Compound 12

**Figure 7:**
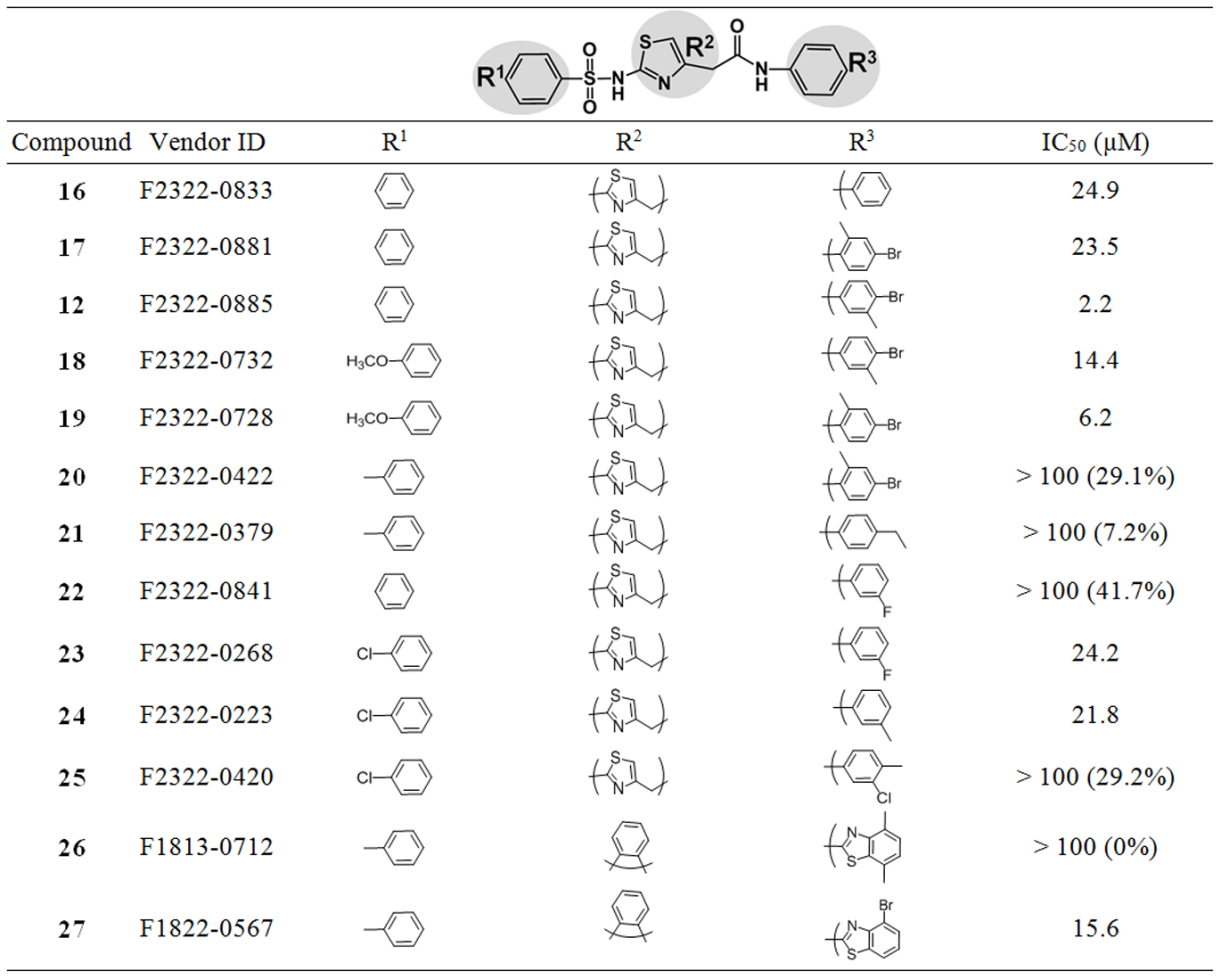
Preliminary Structure and Activity Relationship (SAR) of Compound 12. Out of 25 structurally similar Life Chemicals compounds and 28 additional compounds from a scaffold search based on **12**, % inhibition and IC_50_ values of 13 compounds are shown for comparison. IC_50_ values were determined from two to four independent assays. The percent inhibitions of each compound are shown in parenthesis in cases of IC_50_ value above 100 µM at 50 µM compound concentration.

**Figure 8:**
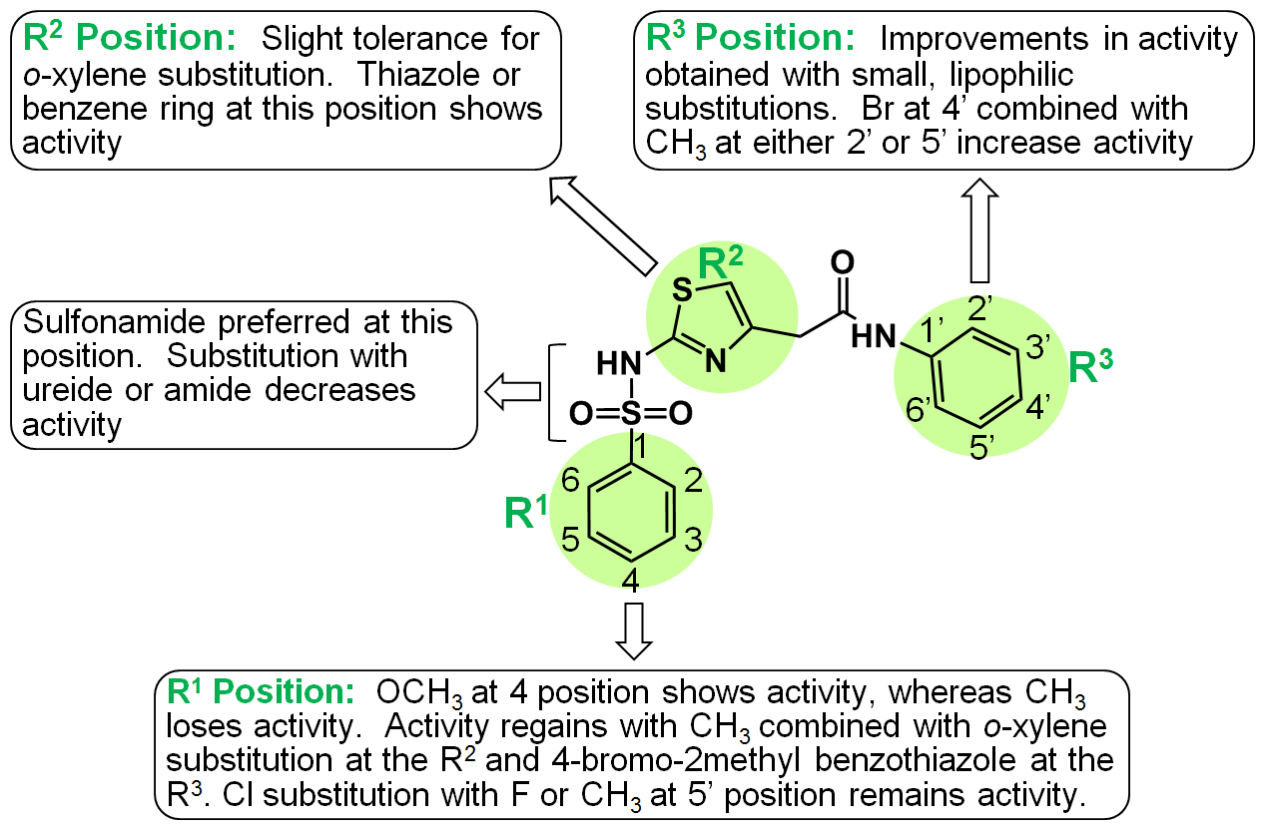
Preliminary Structure and Activity Relationship (SAR) map of compound 12. A total of 53 similarly structured compounds were analyzed to generate this map (See **Figure 7** for structures and IC_50_ values for 13 compounds).

Because our focused Life Chemicals library contains many compounds that are structurally similar, we were able to perform an initial Structure-Activity-Relationship (SAR) analysis with **12**, our best hit. There were 25 compounds with scaffolds similar to **12** in the Life Chemicals library, and 28 additional commercially available compounds were ordered from a scaffold search. Of 53 similarly structured compounds, 18 showed over 50% inhibition at 50 µM concentration. The percent inhibition of the next 20 compounds varied between 30% and 50%, and the rest were below 30% inhibition. We were able to obtain IC_50_ values of the best 18 compounds. A preliminary SAR analysis of compound **12** (**Figure 7**) indicates that the benzene ring with or without additional side-groups at the R1 position showed inhibitory activity. Moderate activity was observed with either a methoxy group (compounds **18** and **19**) or chlorine (**23** and **24**) at the para position of the benzene ring. A methyl at the para position causes complete activity loss when the R2 and R3 structures remain constant (**20** and **21**). However, activity is regained with a methyl at the R1 para position, combined with an *o*-xylene substitution at the R2 position and 4-bromo-2methyl benzothiazole substitution at the R3 (**27**). The R2 position shows a slight tolerance for aromatic group substitution, with activity retained using either 2, 4-disubstituted thiazole (**16**–**25**) or *o*-xylene (**26**–**27**). At the R3 position, a phenyl ring with methyl and bromine substitutions at the meta and para positions (**12**) or a benzothiazole ring shows activity. Of the 53 similar compounds tested, structures and IC_50_ or percent inhibition values of 13 selected compounds are summarized in **Figure 7**, and a preliminary SAR map is shown in **Figure 8**. Compound **12** showed the best inhibitory activity among the 53 tested compounds. In order to complete this SAR map, synthesis of new compounds that will fill the gap is necessary.

### Whole Cell Lysate Inhibition Assay

To test the inhibitory activity under more physiological conditions, we analyzed our best hit compound **12** by a whole cell lysate assay using two HCV subgenomic replicon cell lines, subgenomic replicon 1b (sg1b) and 2a (sg2a), along with a control compound, BILN-2061. EC_50_ values of BILN-2061 with replicon cells 1b and 2a are known to be 3.0 nM and 67 nM, respectively [34], [35], and IC_50_ values from our biochemical assay are 4.6 nM and 12 nM. The IC_50_ value of BILN-2061 increased ∼8-fold and ∼19-fold with the whole cell lysate 1b and 2a, respectively, compared to the original biochemical assay (**Table 4**). The IC_50_ values of **12** increased 10–20 fold with the whole cell lysates of both sg1b and sg2a, but inhibitory activities of this compound were still observed with IC_50_ values below 120 µM with both sg1b and sg2a. As expected, the IC_50_ value of compound **12** against the subgenomic 2a whole cell lysate was much higher than that against genotype 1b since it was ∼6-fold less effective against genotype 2a in the biochemical assay. There are a number of reasons for the increase in IC_50_ values of the compounds in the whole cell lysate assay. The major variable is the differences in the environmental conditions, which include not only differences in salts and buffers, but also competing interaction partners and general accessibility of the enzyme which may artificially aggregate with cellular membranes and lipids after cell lysis. As such, in the whole cell lysate environment, we suspect that the actual working compound concentration may be much lower than that *in vitro*, which could explain the apparent increase in IC_50_ values with both our hit and the control, BILN-2061.

**Table 4:** Inhibitory activity of 12 and a control with HCV subgenomic replicon cell lysates.

Compound	IC_50_ (µM)			
	Purified NS3/4ASub-genotype 1b	HCV repliconcell lysate sg 1b	Purified NS3/4ASub-genotype 2a	HCV repliconcell lysate sg 2a
BILN-2061	0.0046±0.0005	0.037±0.010	0.012±0.001	0.23±0.03
**12**	2.2±0.4	29.3±6.8	12.7±1.2	115±7

All IC_50_ values were determined from three independent assays. sg1b, HCV subgenomic replicon 1b; sg2a, HCV subgenomic replicon 2a.

### Computational Modeling of Hit Compounds

Two hit compounds (**12** and **13**) were competitive inhibitors with respect to the substrate of the NS3 protease according to our mode of inhibition studies. Both compounds were docked into the active site of the NS3 protease (genotype 1b) to investigate their binding modes. The docked poses of **12** and **13** with the highest scores are shown in **Figure 6B**. The sulfonamide group of compound **12** can be seen binding in the catalytic site of the NS3 protease, mimicking the transition state formed by the NS3 protease substrates during cleavage, thereby effectively inhibiting the enzyme. These results correlate well with our activity observations during SAR analysis, which indicated a preference for the sulfonamide group of compound **12** (a potential Sp3 transition state mimic) over amide and ureide groups at this position with respect both to the distance from the core scaffold as well as the unmasked charge. According to these docking predictions, **12** and **13** bind to the same site and overlap well with a known macrocyclic inhibitor (ITMN191) [36].

To further optimize these top two hit compounds, an integrated synthetic strategy is under development that will explore the optimal balance of structural modifications affecting drug resistance, potency, and toxicity based upon the modeled interactions. **12** contains bulky P2 moieties and is therefore susceptible to cross-resistance against mutations at R155 and A156. Substitutions with smaller moieties at this position are planned to investigate their effects on activity against various mutants. To compensate for any loss of binding affinity that may accompany these changes, additional binding interactions can be exploited by building into the S1’–S2’ and S3–S4 sub-sites of the protease active site, currently unoccupied by either inhibitor.

## Conclusions

Herein, we report the discovery of novel, non-peptidic small molecule competitive inhibitors of HCV NS3/4A. The four structurally diverse compound libraries that were screened by HTS produced 15 compounds with IC_50_ values below 50 µM. An enzyme omission assay and a thorough counter-screen by SPR effectively eliminated false positives and led us to discover two hits. The analysis of the mechanism of inhibition showed that both of our small molecule compounds are competitive inhibitors with respect to the NS3 protease substrate. Follow-up studies using two hit compounds with NS3/4A enzymes from four genotypes confirmed cross-genotypic activity of both inhibitors with IC_50_ values below 20 µM against the NS3/4A enzymes from four tested HCV genotypes. Considering the fact that the two FDA-approved NS3 inhibitors are only active against genotype 1, our discovery may provide a route for developing inhibitors with pan-genotypic activity. Most of the NS3 inhibitors in clinical trials and the two FDA-approved compounds show a significant loss of activity upon the development of HCV drug-resistant mutants. Of the two inhibitors we identified, compound **12** maintained its inhibitory activity against five common drug-resistant mutants of full-length NS3/4A from genotype 1b tested in this study. This small molecule inhibitor could potentially remain effective against other drug-resistant mutants as well. In addition, this newly identified compound had comparable activity against both the NS3/4A protease domain alone and the full-length NS3/4A. This result in addition to competitive inhibition provides strong evidence that this small molecule inhibitor binds to the active site of the NS3 protease, rather than to an allosteric site. The lead compound showed selectivity for the HCV NS3/4A serine protease over two human serine proteases, and the HCV replicon cell lysate assay confirmed inhibitory activity in the cellular environment.

To summarize, we have discovered one promising small molecule inhibitor having IC_50_ values below 20 µM against NS3/4As from four HCV genotypes and five common drug-resistant mutants of NS3/4A from genotype 1b. Thorough counter-screens after HTS were used to filter out false positives during hit validation. Enzymatic characterization along with preliminary SAR and docking analyses have given us insight into directions that can be followed to further optimize our lead compound.

## Supporting Information

File S1 **Figure S1, Comparison of HCV NS3 protease and human serine proteases.** (**A**) Sequence aligned scores of 14 human serine proteases with HCV NS3 protease by ClustalW2. (**B**) 3D alignment of the active site of HCV NS3 protease colored in plum and a P2–P4 macrocyclic inhibitor colored in yellow superimposed with trypsin colored in green and chymotrypsin colored in blue. (**C**) 3D alignment of the active site of HCV NS3 protease colored in yellow and 14 human serine proteases. Images were prepared using Chimera v1.6.1, UCSF, 2012 [37]. **Figure S2, Multiple sequence alignment of HCV NS3 and 14 human serine proteases by ClustalW2.** **Table S1,** **MRM transitions, MS parameters and eluent composition during the LC/MS/MS analysis. Table S2, List of the known reactive functionalities and/or known toxicities that were used to filter out compounds to build our Life Chemicals library.**
(DOC)
