# Supplementary material for: High-Throughput Screening (HTS) and Hit Validation to Identify Small Molecule Inhibitors with Activity against NS3/4A proteases from Multiple Hepatitis C Virus Genotypes
Source: PLoS One. 2013 Oct 9;8(10):e75144. doi: 10.1371/journal.pone.0075144 (PMC3793977; doi:10.1371/journal.pone.0075144)
Supplement: File S1 — Figure S1, Comparison of HCV NS3 protease and human serine proteases. (A) Sequence aligned scores of 14 human serine proteases with HCV NS3 protease by ClustalW2. (B) 3D alignment of the active site of HCV NS3 protease colored in plum and a P2–P4 macrocyclic inhibitor colored in yellow superimposed with trypsin colored in green and chymotrypsin colored in blue. (C) 3D alignment of the active site of HCV NS3 protease colored in yellow and 14 human serine proteases. Images were prepared using Chimera v1.6.1, UCSF, 2012 [37]. Figure S2, Multiple sequence alignment of HCV NS3 and 14 human serine proteases by ClustalW2. Table S1, MRM transitions, MS parameters and eluent composition during the LC/MS/MS analysis. Table S2, List of the known reactive functionalities and/or known toxicities that were used to filter out compounds to build our Life Chemicals library. (DOC) [file pone.0075144.s001.doc]

**Supporting information for**

# High-throughput screening (HTS) and hit validation to identify small molecule inhibitors with activity against NS3/4A proteases from multiple Hepatitis C Virus genotypes

Hyun Lee^1^, Tian Zhu^1^, Kavankumar Patel^1^, Yan-Yan Zhang^2^, Lena Truong^1^, Kirk E. Hevener^1,4^, Joseph L. Gatuz ^1^, Gitanjali Subramanya^3,5^, Hyun-Young Jeong^2^, Susan L. Uprichard^3,5^ and Michael E. Johnson^1,*^

^1^ Center for Pharmaceutical Biotechnology and Department of Medicinal Chemistry and Pharmacognosy, University of Illinois at Chicago, Chicago, IL, USA

^2^Department of Pharmacy Practice, University of Illinois at Chicago, Chicago, IL, USA

^3^ Department of Medicine, University of Illinois at Chicago, Chicago, IL, USA

^∗^To whom correspondence should be addressed

Michael E. Johnson: (Phone) 312-996-9114, (Fax) 312-431-9303, (Email) mjohnson@uic.edu

**Footnote**

^4^ Present address: Department of Biomedical and Pharmaceutical Sciences, Idaho State University, Meridian, ID, USA

^5^ Present address: Department of Medicine, Loyola University Medical Center, Maywood, IL, USA

# Contents

1. Supplementary Methods………………………………….…………………………………………..S3

2. Supplementary Figures………………………………………………………………………………..S5

Figure S1. Comparison of HCV NS3 protease and human serine proteases

Figure S2. Multiple sequence alignment of HCV NS3 and 14 human serine proteases

3. Supplementary Tables…………………………………………………………………….……….......S7

Table S1. List of the known reactive functionalities and/or known toxicities that were used to

filter out compounds to build our Life Chemicals library

Table S2. MRM transitions, MS parameters and eluent composition during the LC/MS/MS

analysis

4. NMR Spectra and LC/MS Analysis of Published Compounds………………………………………S10

5. References............................................................................................................................................S15

# 1. Supplementary Methods

## Kinetic parameter determination and assay optimization for HTS.

Assay optimization was done for mainly five factors: substrate concentration, reducing agent effect, enzyme concentration, DMSO tolerance, and enzyme stability. In order to determine the correct substrate concentration for the HTS assay, the Michaelis constant (K_M_) was determined in the presence of each of the four reducing agents and in the absence of reducing agent as described.[^1^](#_ENREF_1) Briefly, the activity of the NS3/4A complex was measured by continuous kinetic assay with a FRET-based substrate Ac-DE-Dap(QXL520)-EE-Abu-ψ-[COO]AS-C(5-FAMsp)-NH_2_ (Anaspec). The HCV NS3/4A assay was performed in the assay buffer containing 50 mM Tris, pH 7.6, 0.5% Chaps, 20% glycerol, 2 mM GSH, and 0.01 mg/mL BSA with 20 µL total assay volume in 384-well black low volume microplates (Corning Inc.). A series of substrate concentrations (0 to 50 µM) was prepared, and the enzyme reaction was initiated by adding HCV NS3/4A (10 nM final concentration). The same series of substrate concentrations without any enzyme was also measured as a control. Fluorescence intensity (492/520 nm, excitation/emission) was monitored continuously for 10 minutes with a POLARstar OPTIMA microplate reader (BMG LABTECH). The Michaelis constant (K_M_) and maximal activity (V_max_) were calculated by fitting the data with the hyperbolic equation (1) where y is initial velocity and x is the concentration of substrate.


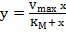
 ..........................................................(1)

Once the optimal substrate concentration was determined, the NS3/4A enzyme concentration was optimized by titration of varying enzyme concentrations to obtain a linear reaction curve for at least 30-60 minutes. The 10 nM final concentration of NS3/4A enzyme produced a linear initial velocity curve for 60 minutes and also gave a good Z-factor range for HTS.

A series of DMSO concentrations ranging from 0 to 10% was tested for enzyme activity and substrate stability. Less than 10% inhibition of enzyme activity was observed with up to 8% DMSO, and the substrate of NS3/4A showed sensitivity to DMSO starting at 5%. Therefore, less than 4% final DMSO concentration was used for all assays.

# 2. Supplementary Figures

A B

| Enzyme | Uniprot ID | PDB  Code | Sequence Aligned Score  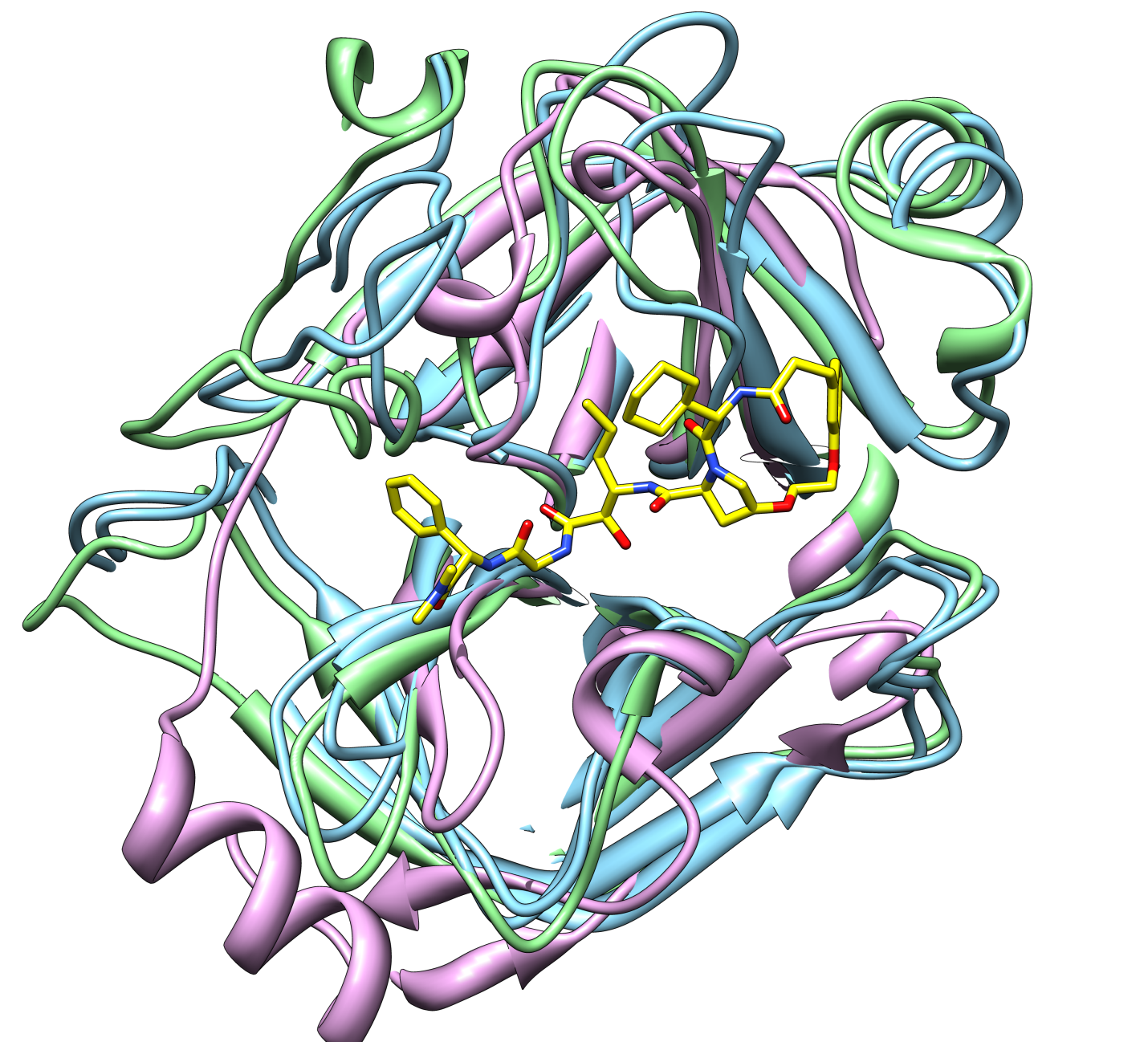 | | |
| --- | --- | --- | --- | --- | --- |
|  |  |  | HCV NS3 Protease | Trypsin | Chymotrypsin |
| HCV NS3 Protease | Q0ZMF9 | 2GVF | 100 | 9 | 13 |
| Chaymase | P23946 | 1KLT | 8 | 33 | 31 |
| Factor VIIa | P08709 | 2FLR | 11 | 36 | 31 |
| Factor Xa | P00742 | 2Y5F | 2 | 34 | 28 |
| Kallikrein | P49862 | 2QXG | 3 | 40 | 34 |
| Plasmin | P00747 | 1DDJ | 12 | 40 | 36 |
| Urokinase | P00749 | 3QN7 | 12 | 35 | 32 |
| Thrombin | P00734 | 3U8O | 10 | 35 | 34 |
| tPA | P00750 | 1BDA | 10 | 33 | 32 |
| Trypsin | P07477 | 1TRN | 9 | 100 | 39 |
| Tryptase | P20231 | 3V7T | 3 | 35 | 33 |
| Human Elastase | P08246 | 1B0F | 13 | 29 | 25 |
| Cathepsin G | P08311 | 1AU8 | 12 | 34 | 28 |
| Hepsin | P05981 | 3T2N | 15 | 36 | 35 |
| Chymotrypsin | P17538 | NA | 13 | 39 | 100 |

**C**


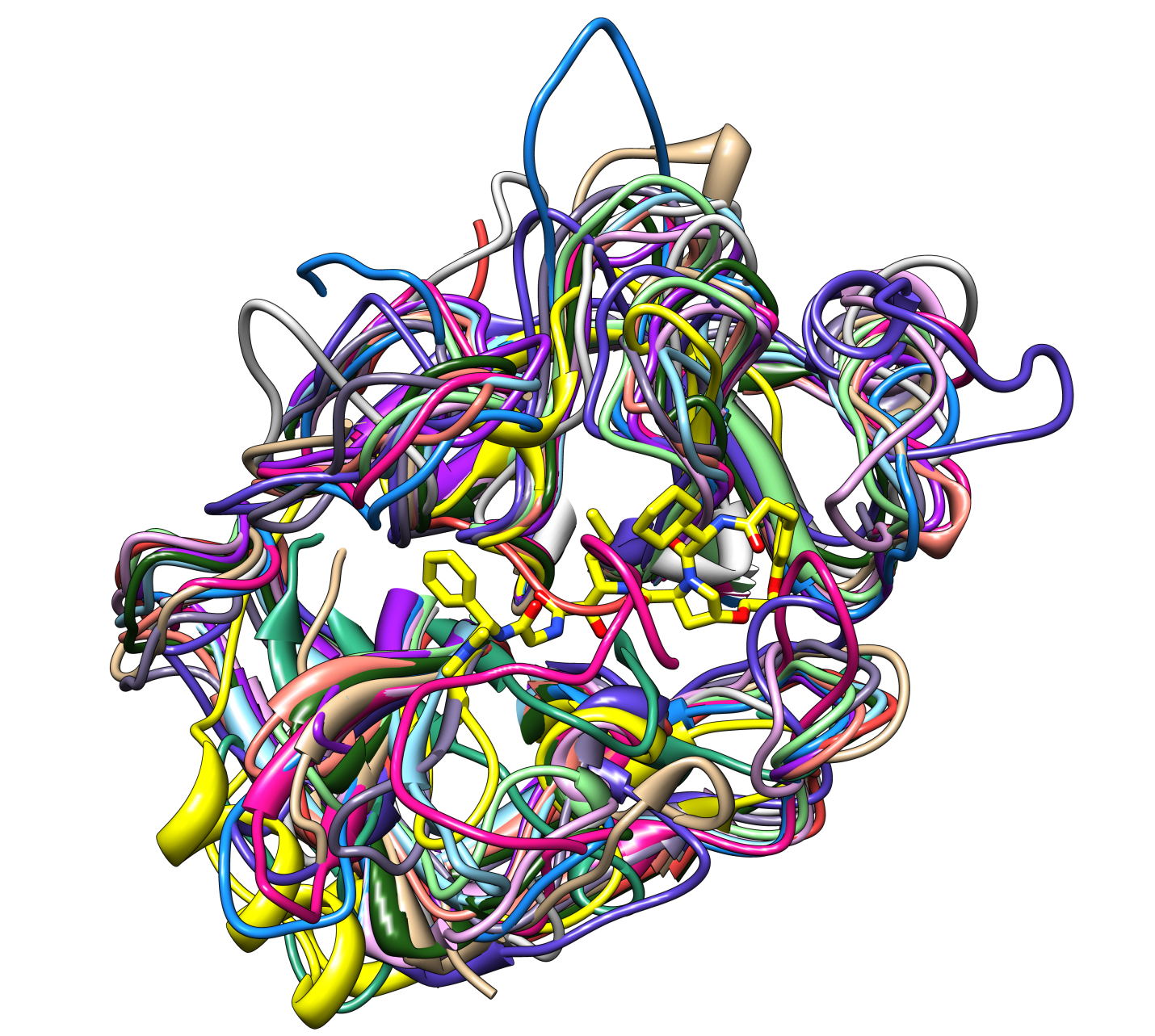


**Figure S1. Comparison of HCV NS3 protease and human serine proteases.** (A) Sequence aligned scores of 14 human serine proteases with HCV NS3 protease by ClustalW2. (B) 3D alignment of the active site of HCV NS3 protease colored in plum and a P2-P4 macrocyclic inhibitor colored in yellow superimposed with trypsin colored in green and chymotrypsin colored in blue. (C) 3D alignment of the active site of HCV NS3 protease colored in yellow and 14 human serine proteases. Images were prepared using Chimera v1.6.1, UCSF, 2012.[^2^](#_ENREF_2)


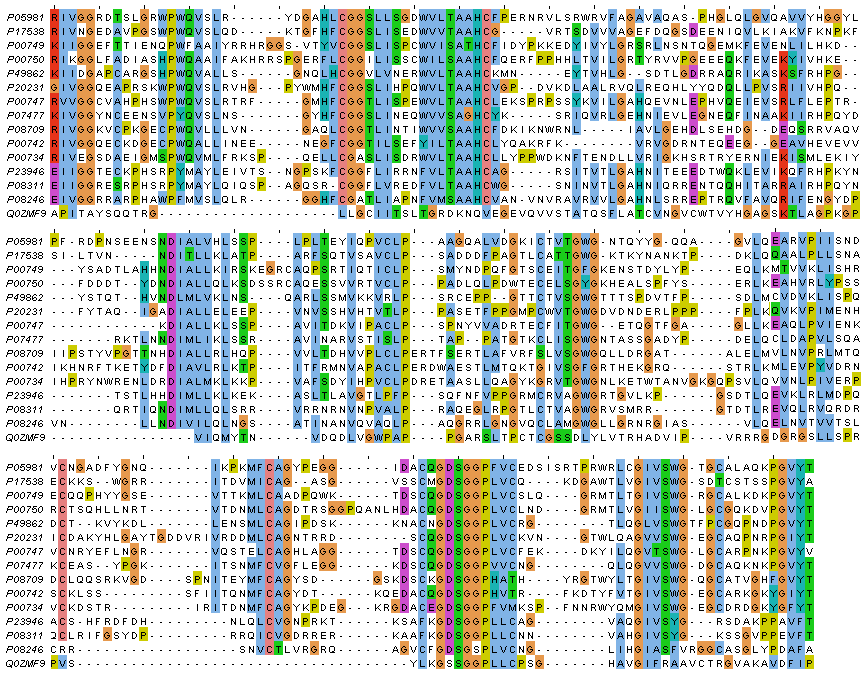


**Figure S2.**  Multiple sequence alignment of HCV NS3 and 14 human serine proteases by ClustalW2.

# 3. Supplementary Tables

# Table S1. List of the known reactive functionalities and/or known toxicities that were used to filter out compounds to build our Life Chemicals library.

**A. Potentially Reactive Groups**


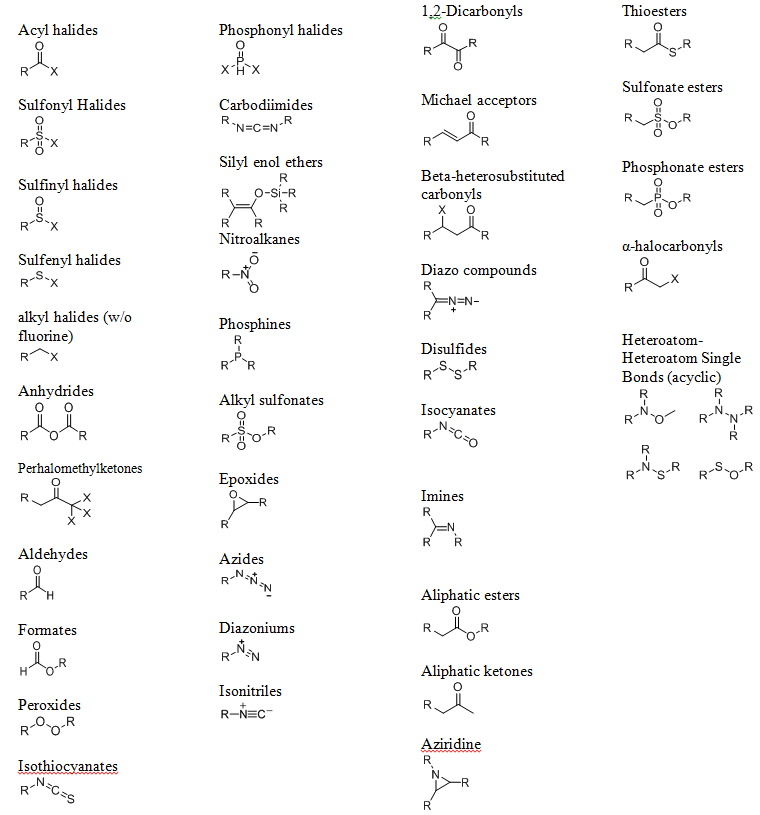


**B. Potentially Toxic Groups**

**
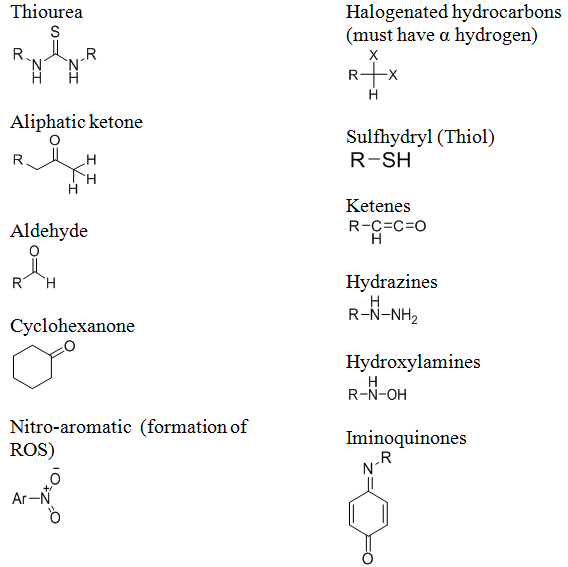
**

**Table S2.** MRM transitions, MS parameters and eluent composition during the LC/MS/MS analysis.

| Compounds | MRM | Declustering | Collision | Collision Cell | Mobile Phase |
| --- | --- | --- | --- | --- | --- |
|  | Transition | Potential (V) | Energy (eV) | Exit Potential (V) | %B (min) |
| 4 | 445.2→88.2 | 81 | 37 | 4 | 5(0)→5(1)→90(2)→90(3)→5(3.5)→5(10) |
| 12 | 466.0→253.2 | 66 | 29 | 4 | 20(0)→90(1)→90(2)→20(2.5)→20(10) |
| Phenytoin | 253.3→182.2 | 32 | 24 | 3 | Internal Standard |

4**. NMR Spectra and LC/MS Analysis of Published Compounds**

There were two confirmed hits (Compound **12** and **13**) in this study. Both compounds were repurchased from Life Chemicals. The purity of the compounds has been determined by LC-MS, HPLC and/or NMR to be ≥99%.

Compound **12** (F2322-0885)

**Data from the vendor:**


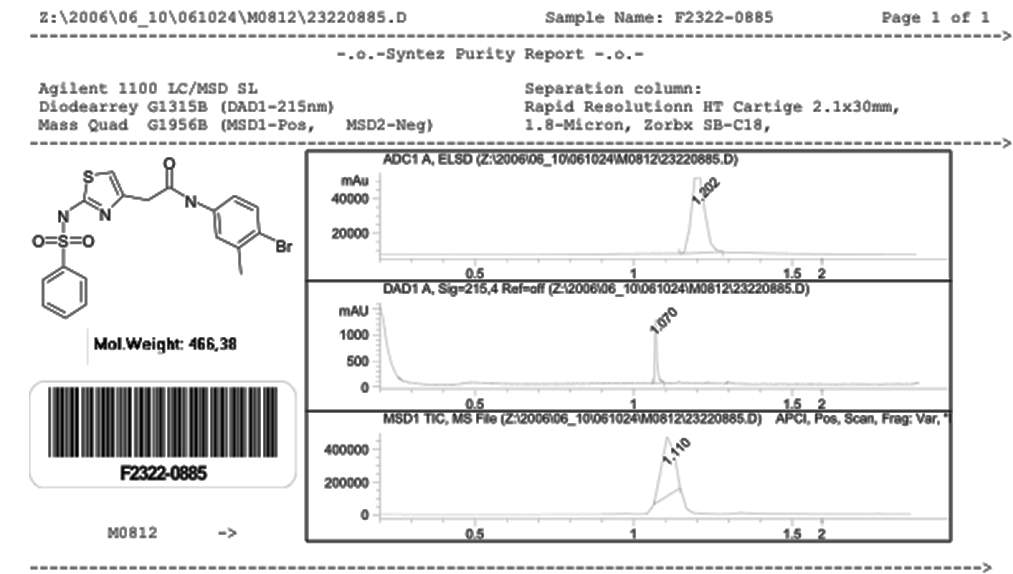

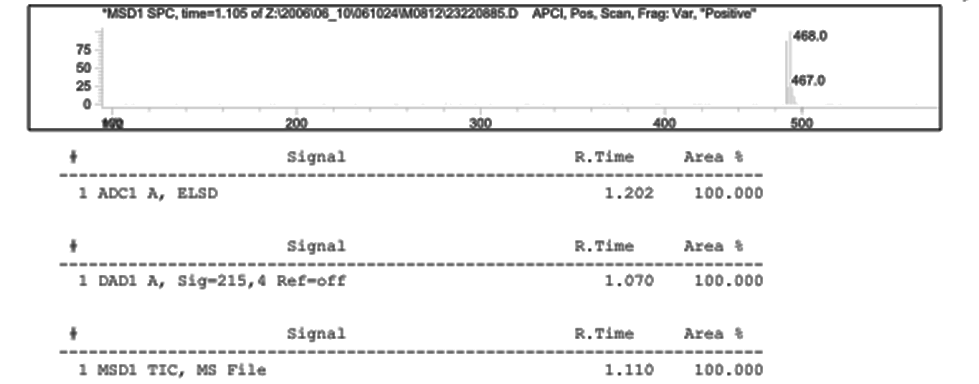


**Data obtained at UIC:**


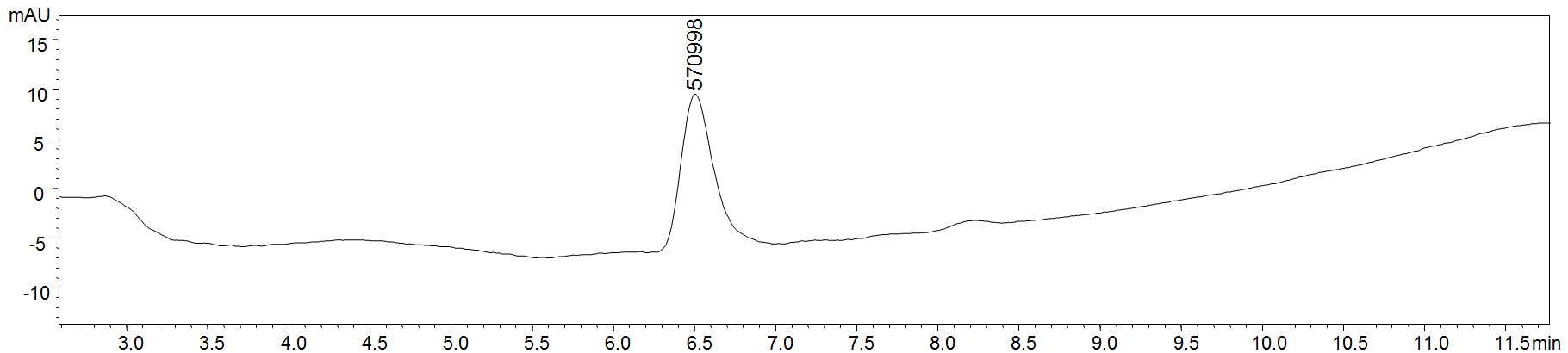

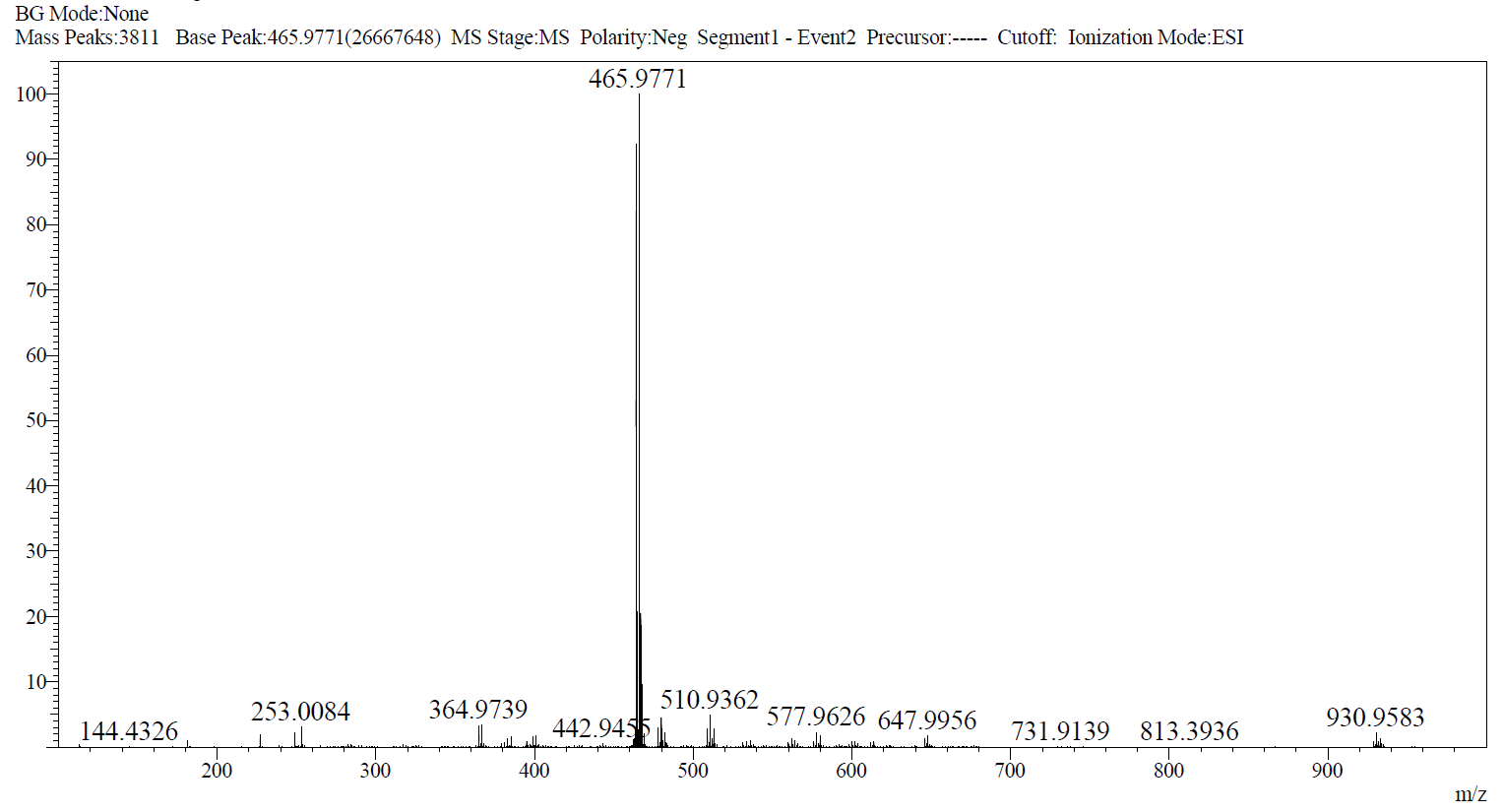

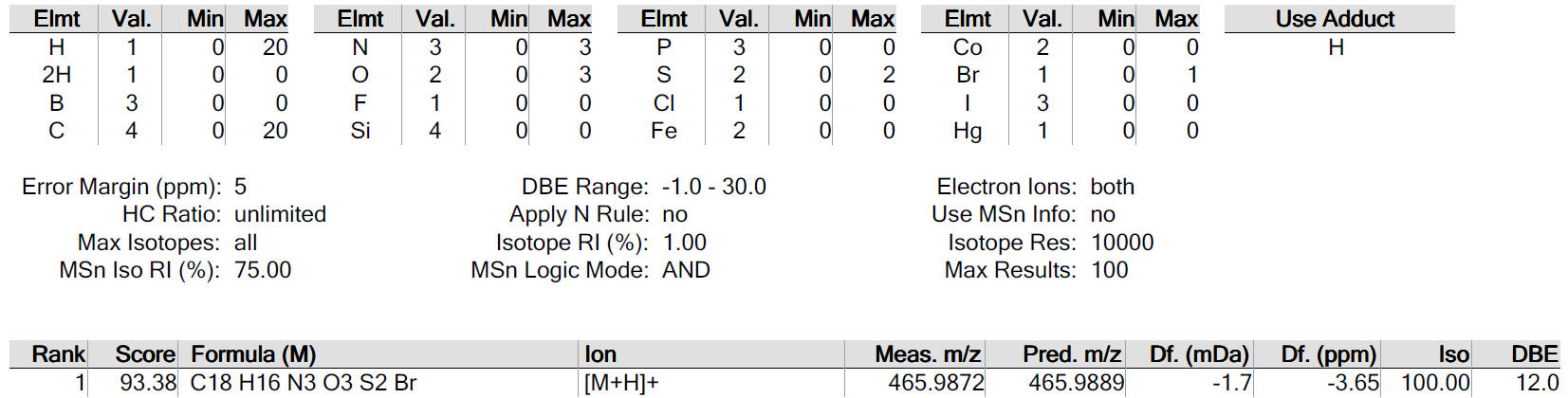


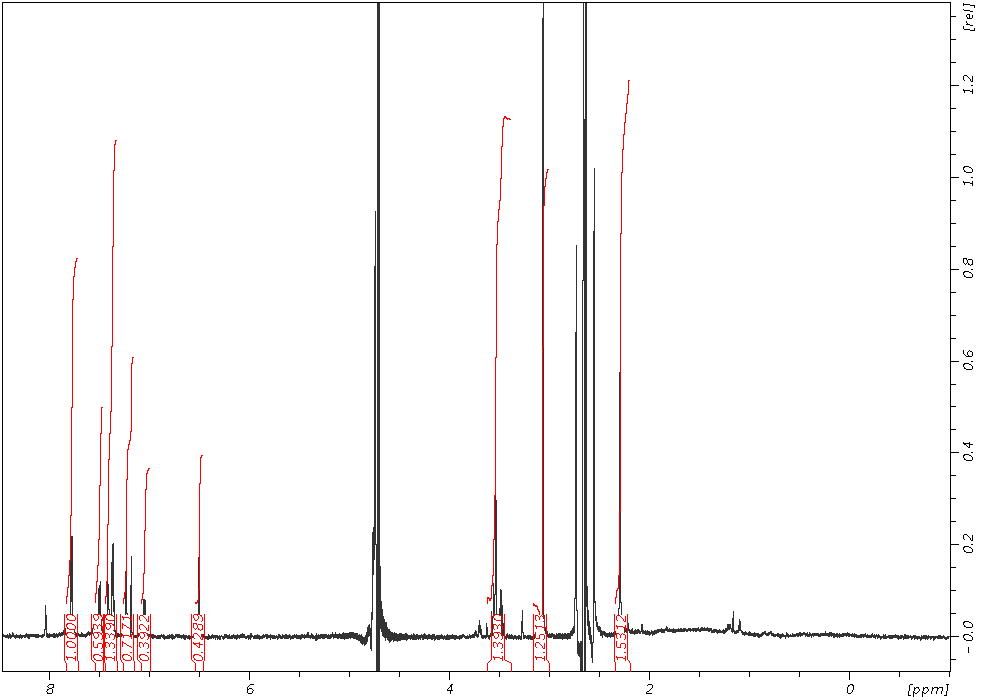


Water DMSO double solvent suppression in 50 mM PBS pH 7.5

**HPLC purification of compound 12 after purchase:**

Column used: YMC-Pack ODS-AQ 250 x 20 mm I.D. S-5 micrometer

Gradient condition: 0~30 min MeOH 90%-water 10% to MeOH 100%

30~50 min MeOH 100%

Fractions #32 - #36 were collected, and all solvent was removed using vacuum speedvac.


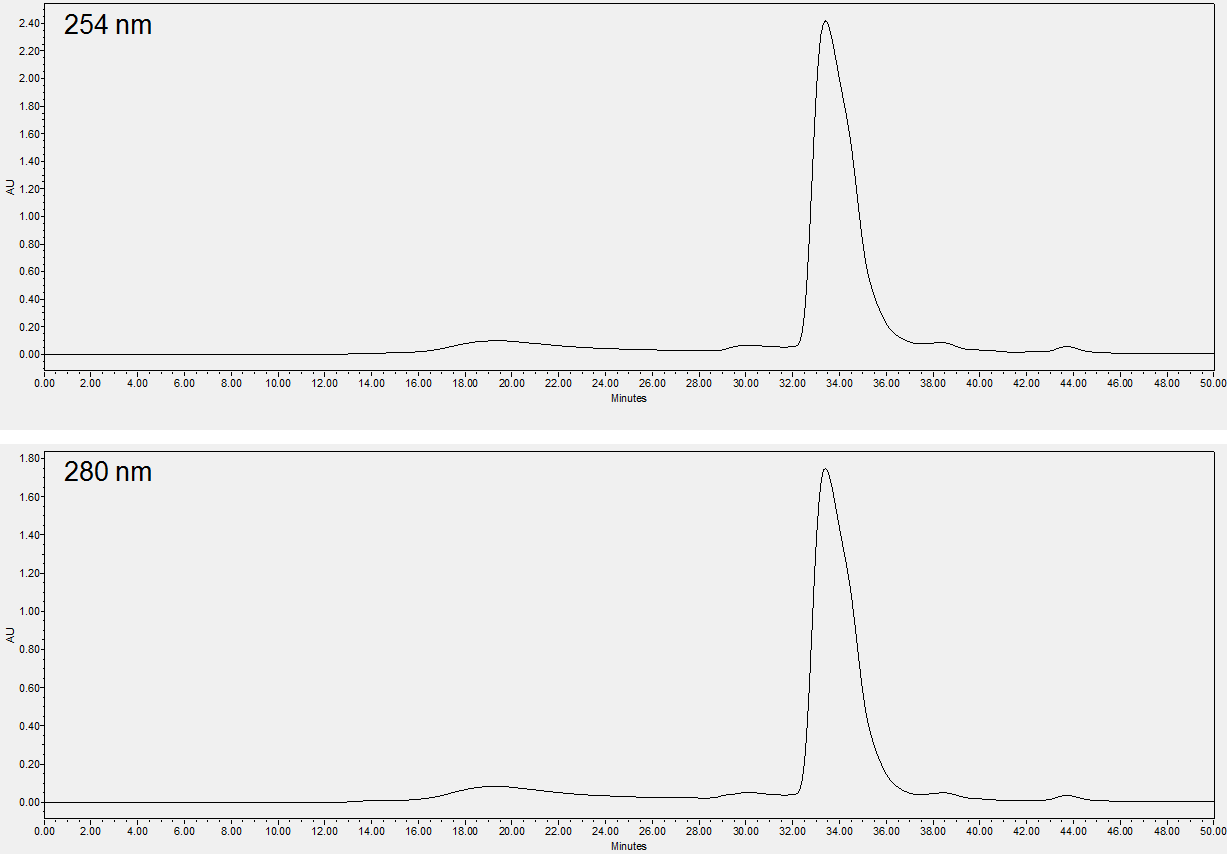


Compound **13** (F2416-0384)

**Data from the vendor:**


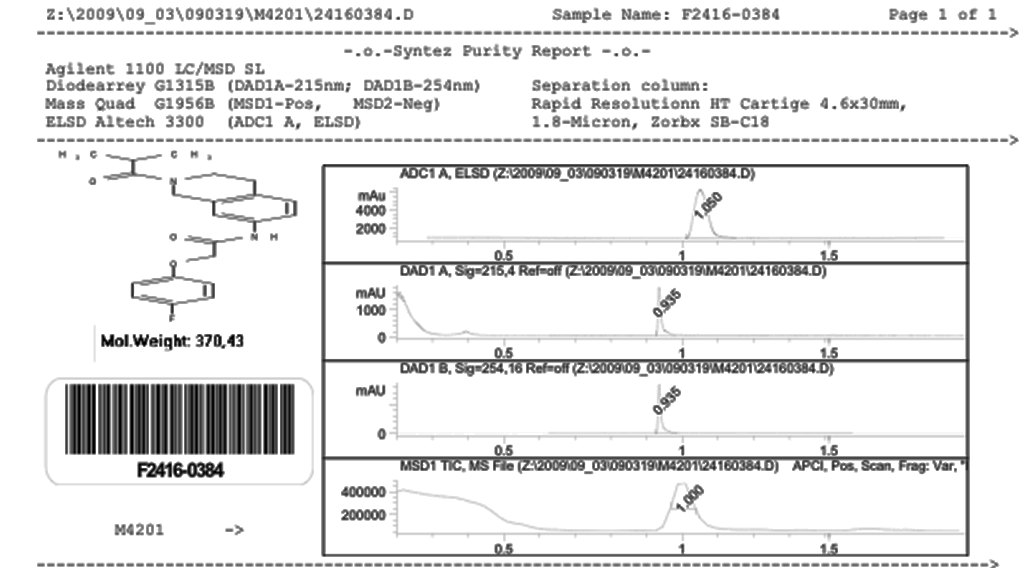

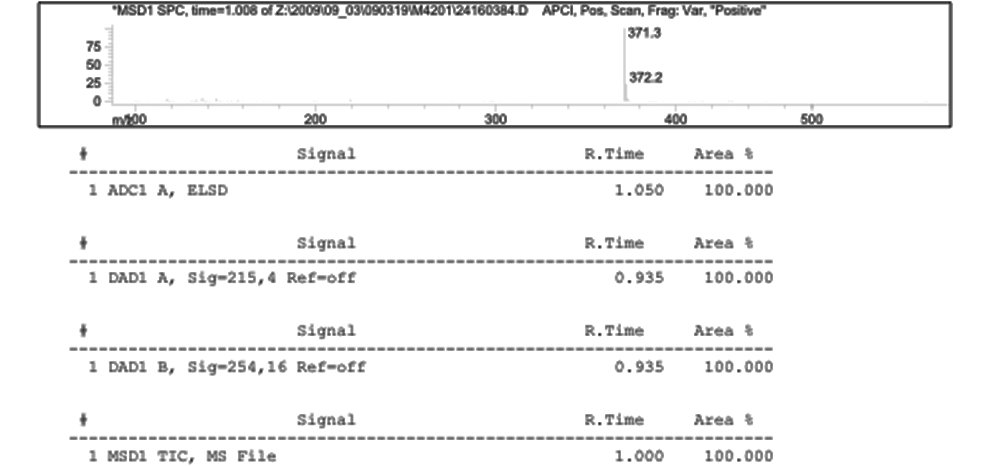


**Data obtained at UIC :**

**
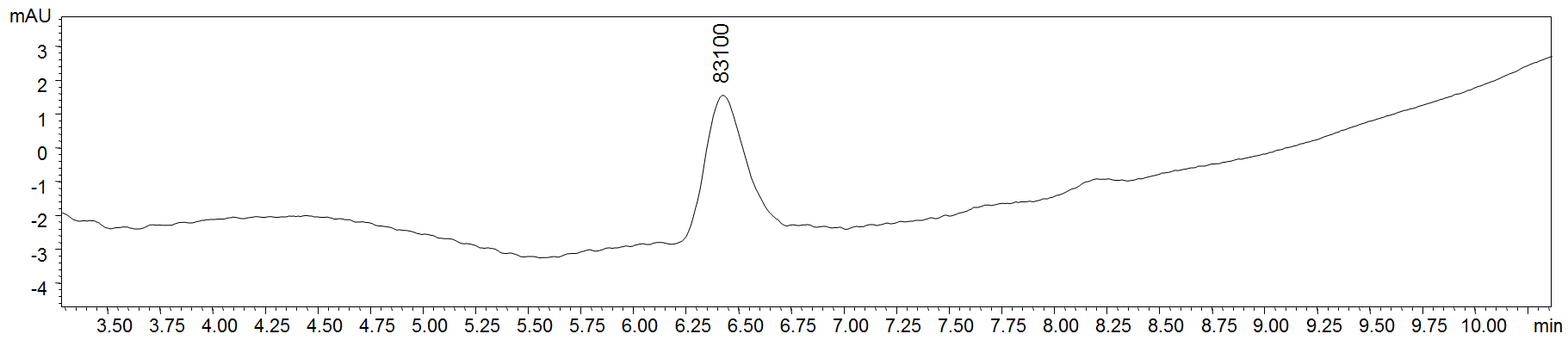

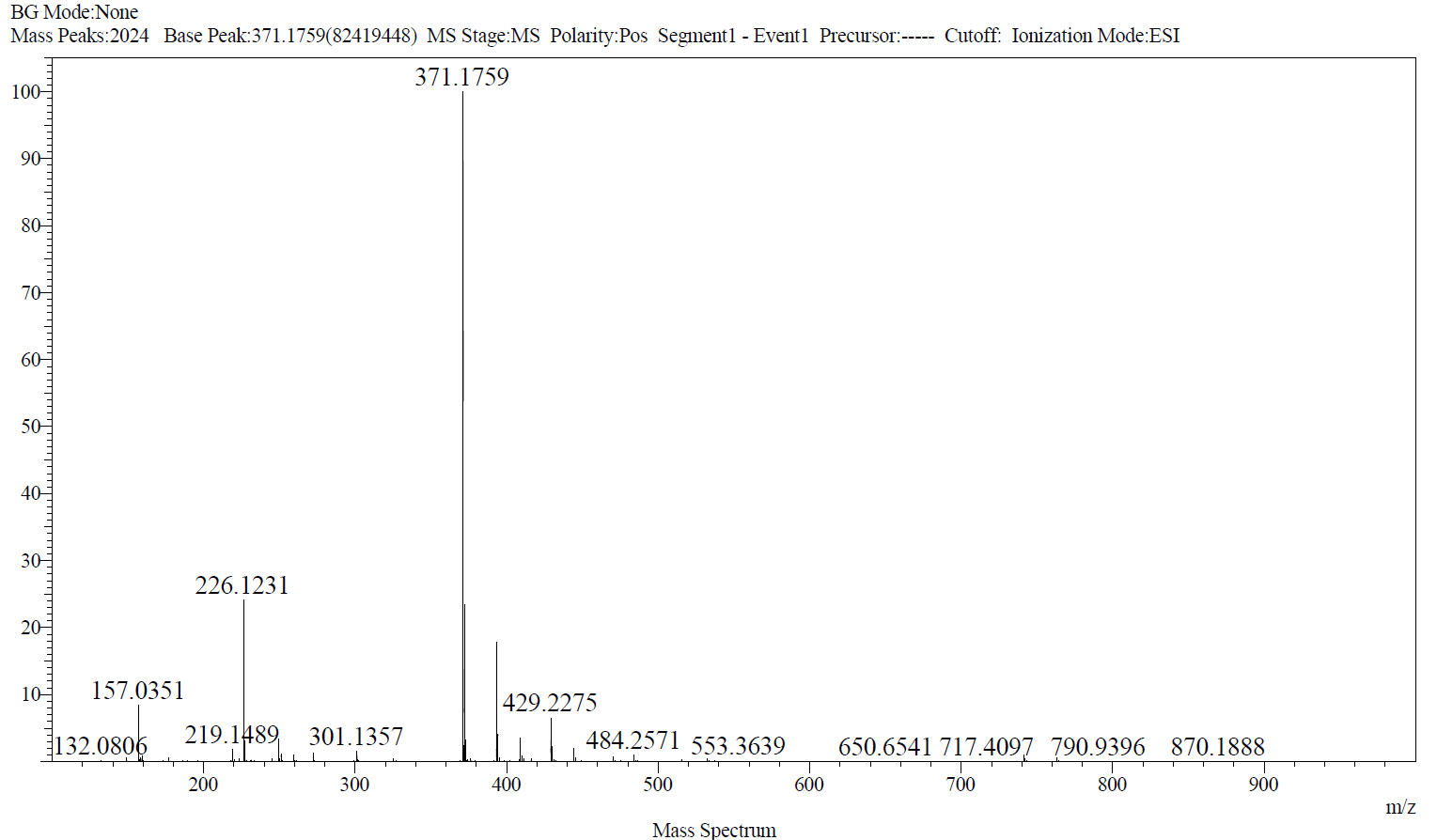

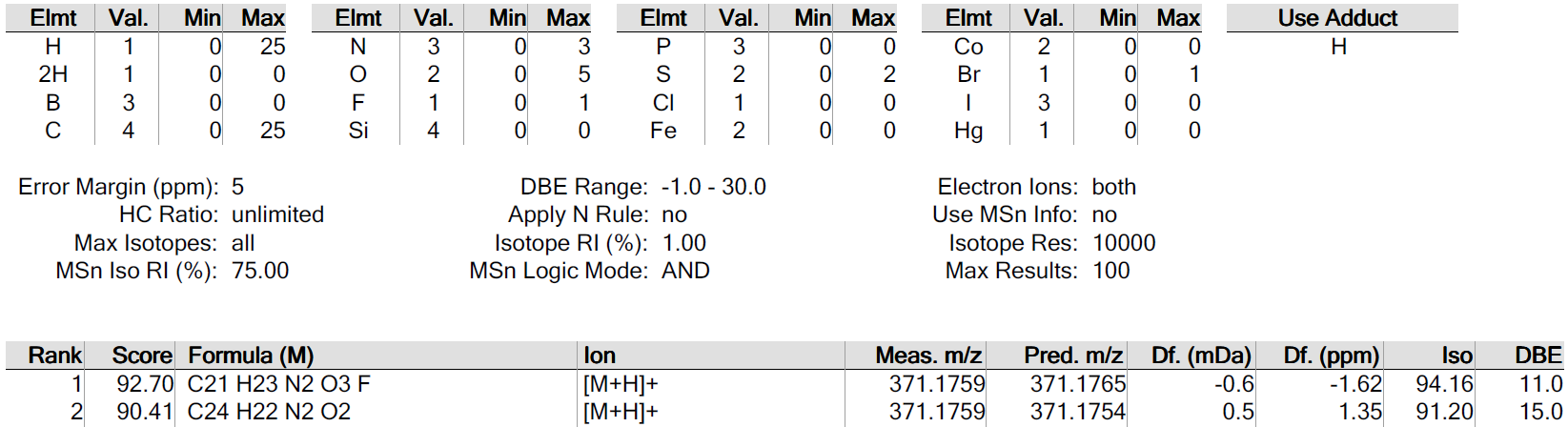
**


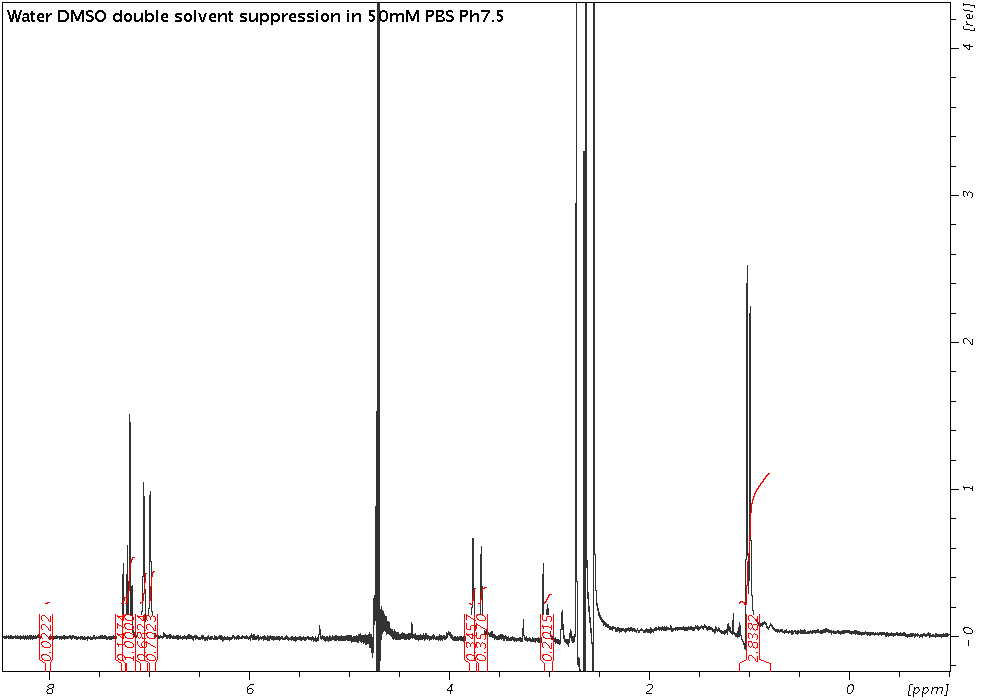


**References**

1. Lee, H.; Torres, J.; Truong, L.; Chaudhuri, R.; Mittal, A.; Johnson, M. E. Reducing agents affect inhibitory activities of compounds: Results from multiple drug targets. *Anal Biochem* **2012,** 423, 46-53.

2. Pettersen, E. F.; Goddard, T. D.; Huang, C. C.; Couch, G. S.; Greenblatt, D. M.; Meng, E. C.; Ferrin, T. E. UCSF Chimera--a visualization system for exploratory research and analysis. *J Comput Chem* **2004,** 25, 1605-12.
